# Distinct and redundant roles for zebrafish *her* genes during mineralization and craniofacial patterning

**DOI:** 10.3389/fendo.2022.1033843

**Published:** 2022-12-12

**Authors:** Amanda Stenzel, Abigail Mumme-Monheit, Juliana Sucharov, Macie Walker, Jennyfer M. Mitchell, Bruce Appel, James T. Nichols

**Affiliations:** ^1^ Department of Craniofacial Biology, University of Colorado-Anschutz Medical Campus, Aurora, CO, United States; ^2^ Department of Pediatrics, Section of Developmental Biology, University of Colorado-Anschutz Medical Campus, Aurora, CO, United States

**Keywords:** craniofacial, skeleton, *her*, mineralization, Notch, bone, osteoblast, patterning

## Abstract

The Notch pathway is a cell-cell communication system which is critical for many developmental processes, including craniofacial development. Notch receptor activation induces expression of several well-known canonical targets including those encoded by the *hes* and *her* genes in mammals and zebrafish, respectively. The function of these genes, individually and in combination, during craniofacial development is not well understood. Here, we used zebrafish genetics to investigate *her9* and *her6* gene function during craniofacial development. We found that *her9* is required for osteoblasts to efficiently mineralize bone, while cartilage is largely unaffected. Strikingly, gene expression studies in *her9* mutants indicate that although progenitor cells differentiate into osteoblasts at the appropriate time and place, they fail to efficiently lay down mineralized matrix. This mineralization role of *her9* is likely independent of Notch activation. In contrast, *her9* also functions redundantly with *her6* downstream of Jagged1b-induced Notch activation during dorsoventral craniofacial patterning. These studies disentangle distinct and redundant *her* gene functions during craniofacial development, including an unexpected, Notch independent, requirement during bone mineralization.

## Introduction

### Notch signaling has diverse functions

The Notch signaling pathway is conserved across metazoans. Notch signaling is highly pleiotropic and functions in the development, homeostasis and regeneration of many different cells and tissues, including skeletal cells (1–3). During canonical vertebrate Notch activation, membrane-bound Jagged or Delta ligands bind to membrane-bound Notch receptors to induce cell-cell communication (4). After binding, the Notch receptor is cleaved releasing the Notch intracellular domain (NICD) which translocates to the nucleus where it directly induces transcription of downstream target genes (5). Canonical Notch downstream targets include the homologs of the Drosophila genes hairy and enhancer of split. In mammals these genes are called *hairy and enhancer of split* (*hes*) (6). In zebrafish the homologous genes are called *hairy and enhancer of split related* (*her*) (7). The *hes* and *her* genes belong to a family of helix-loop-helix transcriptional repressors with Orange domain (8). The roles that these effectors play in various Notch signaling contexts are not yet clear. Further confounding our understanding, some *her* genes (like *her9*) have both Notch-dependent and Notch-independent expression in different situations (9–12). Thus, more work is needed to understand specific roles of the different *hes*/*her* genes, individually and in combination, and to explore contexts where their expression can be Notch dependent or independent.

### Notch functions in craniofacial patterning

The vertebrate craniofacial skeleton is largely derived from post-migratory neural crest cells. After these cells migrate into the pharyngeal arches, various signaling pathways act upon them to confer dorsal versus ventral cellular identity. These dorsal and ventral cells then give rise to the diversely shaped cartilages and bones of the jaw and jaw support structures. The Notch pathway is one signaling system that functions during craniofacial development to specify dorsal versus ventral identity in zebrafish (1, 13–16). Much of our understanding of this process comes from studies of genetic mutants, overexpression conditions, and pharmacological inhibitors in zebrafish. In particular, the Notch ligand *jagged1b* (*jag1b*) is critical for zebrafish craniofacial development, and *jag1b* mutant phenotypes are phenocopied by the Notch cleavage inhibitor dibenzazepine (DBZ) (13). In *jag1b* mutants, dorsal craniofacial structures are shaped more like ventral structures suggesting identity transformations. In support, in *jag1b* mutants, expression of ventral identity genes like *dlx5a* are expanded into dorsal domains (1). These zebrafish studies offer insight into the mechanisms behind human craniofacial disease phenotypes. Humans with mutations in the human ortholog *JAG1* develop Alagille syndrome which is characterized by craniofacial abnormalities including a broad forehead, deep set eyes, small pointed chin, and midface hypoplasia (17). Midface hypoplasia, characterized by a reduction of bone, is observed in both humans and mouse models (18, 19). This dorsal bone reduction defect is similar to the reduced dorsal bone observed in zebrafish *jag1b* mutants. Thus, genetic studies in zebrafish inform the etiology of human genetic disease phenotypes.

Jag1b appears to be the most critical Notch ligand for zebrafish craniofacial development. In contrast, the roles of the various Notch target genes in craniofacial patterning are less clear. For example, *her6* expression is regionalized to the dorsal domain of the pharyngeal arches, making it a strong candidate for functioning downstream of *jag1b* during craniofacial patterning. However, zebrafish *her6* mutants are indistinguishable from wild types (14), indicating *her6* function is not required for dorsal identity patterning, or may function redundantly with other genes. Mice mutant for *Hes1*, the rodent ortholog of *her6*, develop dramatic craniofacial phenotypes (20) at least in part due to neural crest cell survival defects (21). To our knowledge, a patterning function of *Hes1* has not yet been demonstrated in mice. Another Notch target gene of this same family, *her9*, was reported to have overt craniofacial defects when mutagenized in zebrafish (22), but details of these defects were not analyzed, and no figures illustrating the skeletal phenotype were provided. Thus, the role of this gene in craniofacial development remains unknown. However, the human ortholog, *HES4*, is contained within a region deleted in chromosome 1p36 deletion syndrome, which is associated with a small, short and wide head, vision and hearing problems, abnormalities of the skeleton as well as abnormalities of the brain (23). Because there are many genes encompassed by this deletion, whether *HES4/her9* is involved in craniofacial patterning remains unknown. Adding to the confusion, there is no *HES4*/*her9* ortholog in the mouse genome. Therefore, the mouse system cannot inform the function of this gene.

Given that *her6* is specifically expressed in cranial neural crest cells from the dorsal pharyngeal arches, and that *her6* mutants do not develop overt phenotypes in zebrafish, we hypothesized that *her6* functions redundantly with other *her* genes downstream of *jag1b* during zebrafish craniofacial patterning. Double and triple mutant genetic experiments are needed to test this hypothesis.

### Notch target genes regulate skeletal cell differentiation

The Notch pathway also plays a vital role in skeletal development occurring after craniofacial patterning. After regional identity is specified, precursor cells differentiate into chondrocytes and osteoblasts that make cartilage and bone, respectively. Numerous studies implicate Notch signaling and Notch target genes in both chondrocyte and osteoblast cell differentiation (3). Chondrogenic differentiation assays in human cells indicate that HES/HER proteins bind to *SOX9* binding sites in *COL2A1* enhancers, thereby suppressing expression of genes critical for chondrocyte differentiation (24). Similarly, in mouse cells these genes suppress *Sox9* and *Col2a1* expression and chondrogenesis (25). Thus, Notch activation decreases chondrogenesis, at least in these experimental contexts. The role of Notch target genes during osteoblast differentiation is complicated and unclear. Specifically, some studies indicate Notch target genes, including *HES4/her9*, function to promote osteoblast formation (26). Meanwhile others indicate that Notch signaling, *via hes/her* genes, represses osteoblast differentiation (27, 28). Thus, *hes/her* genes are reported to have both an inductive and a suppressive effect on osteoblast differentiation. However, several of these studies were carried out with cell culture overexpression conditions, which may not inform *in vivo* function. A more complete understanding of the complicated role of these genes during osteoblast differentiation will likely arise from studies examining *in vivo* genetic models.

Here we used zebrafish genetics to discover that *her9* function is crucial for osteoblasts to efficiently mineralize bone. Surprisingly, osteoblasts differentiate in the correct time and place in *her9* mutants, yet mineralized matrix production is dramatically impaired. This mineralization role is independent of Notch cleavage. However, we did find a redundant role for *her9* and *her6* downstream of Jagged induced Notch activation during craniofacial patterning.

## Results

### Gene expression suggests *her9* and *her6* function downstream of *jag1b* during craniofacial development

**Figure 1:**
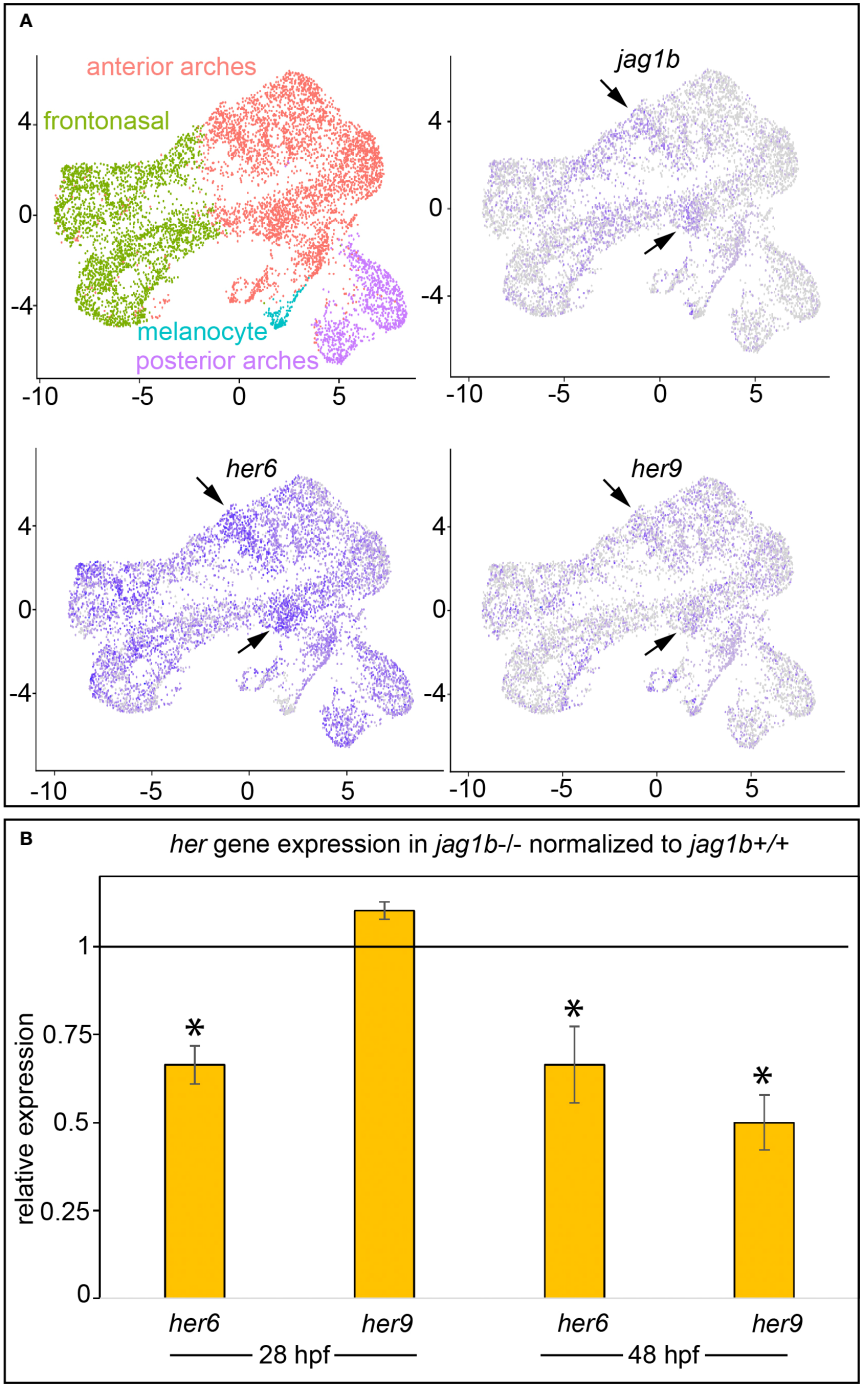
*her6* and *her9* are expressed in the same subpopulation of cranial neural crest cells as *jag1b*, and their expression is dependent upon *jag1b* function. **(A)** UMAPs from single cell RNA-sequencing on 24 hpf sorted cranial neural crest cells. As in our previous work (29), cranial neural crest cells can be clustered into four distinct populations. Based on our previous study and the landmark genes enriched in each population we can identify where the cells reside in the intact animal. Arrows indicate *jag1b* enrichment in a subset of anterior arch cells. Although *her6* is broader, arrows indicate that some of the strongest expressing cells are the *jag1b* enriched populations. *her9* is also broadly expressed among cranial neural crest cells, but at lower levels than *her6*. Arrows indicate *her9* enrichment in the *jag1b*;*her6* expressing populations. **(B)** We performed qPCR on 28 and 48 hpf genotyped heads from wild type and *jag1b* homozygous mutant siblings. Expression of each *her* gene was normalized to *rps18*, then normalized to the relevant wild-type control. Reactions were performed in technical triplicate and the results represent three to six biological replicates. Each biological replicate was pooled heads from 5–6 genotyped homozygous wild types or homozygous mutants. Error bars are standard deviation. Asterisks indicate significant differences from wild type controls at p < 0.05 by students t-test.

*jag1b* functions in cranial neural crest cells of the dorsal anterior pharyngeal arches (arches 1 and 2) (1). We, and others, demonstrated that developing zebrafish heads express *her6*, in a *jag1b* dependent manner (14, 15). Yet, *her6* mutants do not produce overt craniofacial phenotypes (14). These results motivate the hypothesis that *her6* functions redundantly with other *her* genes downstream of *jag1b*. To explore what other *her* genes might be acting downstream of *jag1b* during craniofacial development, we searched for genes expressed in the same cranial neural crest cell population as *jag1b* and *her6*. We examined a 24 hours post fertilization (hpf) single cell RNA sequencing (scRNA-seq) dataset from sorted cranial neural crest cells. This dataset is an unpublished replicate to test for reproducibility of our previously published experiment (29). Both replicates are highly similar, and the results of this comparison will be reported elsewhere. We found that 16 *her* genes are detectibly expressed in this dataset (**Figure S1**), however *her9* and *her6* are the family members with the strongest expression in the skeletogenic, pharyngeal arch neural crest cells. In contrast, many of the other *her* genes are only weakly, or not detectably, expressed in the anterior pharyngeal arches. In fact, most of the other *her* genes in this dataset are restricted to the melanocyte population. Further examining *her9* and *her6* revealed that they are expressed in a subpopulation of anterior arch cranial neural crest cells along with *jag1b* (**Figure 1A**). While *her6* is broadly expressed among cranial neural crest cells, the *jag1b* expressing population is enriched for *her6*. *her9* is broadly, but more weakly, expressed in most cranial neural crest cells but the strongest expression is in the *jag1b* population. We further analyzed these expression data with side-by-side comparisons in two-color plots to look for co-expression between *jag1b*, *her9*, and *her6* (**Figure S2**). These plots further support our interpretation that *jag1b*, *her9* and *her6* are expressed in the same population, and in some cases in the same cells. We next re-clustered the anterior arch population to further analyze *jag1b* and *her9* and *her6* in these cells which give rise to the craniofacial skeleton. In these analyses we include UMAP plots with the expression of known “landmark” genes. These include the pan-anterior arch gene *dlx2a*, the intermediate and ventral anterior arch gene *dlx5a* and the extreme ventral anterior arch gene *hand2*. We observed in these studies, that *jag1b*, known to be expressed in the dorsal anterior arch population (1), is complementary to *dlx5a*, as expected. Moreover *jag1b* expression overlaps with *her9* and *her6* in these analyses. These data further support the finding that *her9* and *her6* are expressed in the same population of cells as *jag1b*. These results make *her9* and *her6* strong candidates for genes functioning downstream of *jag1b* during craniofacial development. To directly test if *her9* is dependent upon *jag1b* function, we performed RT-qPCR in wild types and *jag1b* mutants at 28 and 48 hpf. We previously demonstrated that *her6* is a transcriptional target of *jag1b* (15); expression is downregulated in *jag1b* mutants compared with wild-type controls at both 28 and 48 hpf (**Figure 1B**). Here, we find that *her9* expression is significantly decreased in *jag1b* mutants at 48 hpf compared with wild types. There is no significant difference at 28 hpf. These findings indicate that *her9* expression is *jag1b* independent at 28 hpf and *jag1b* dependent at 48 hpf. That both *her6* and *her9* expression are dependent upon *jag1b* in zebrafish heads at 48 hpf, motivates the hypothesis that they function redundantly downstream of Jag-induced Notch signaling at this stage.

### *her9* is required for timely bone mineralization

**Figure 2:**
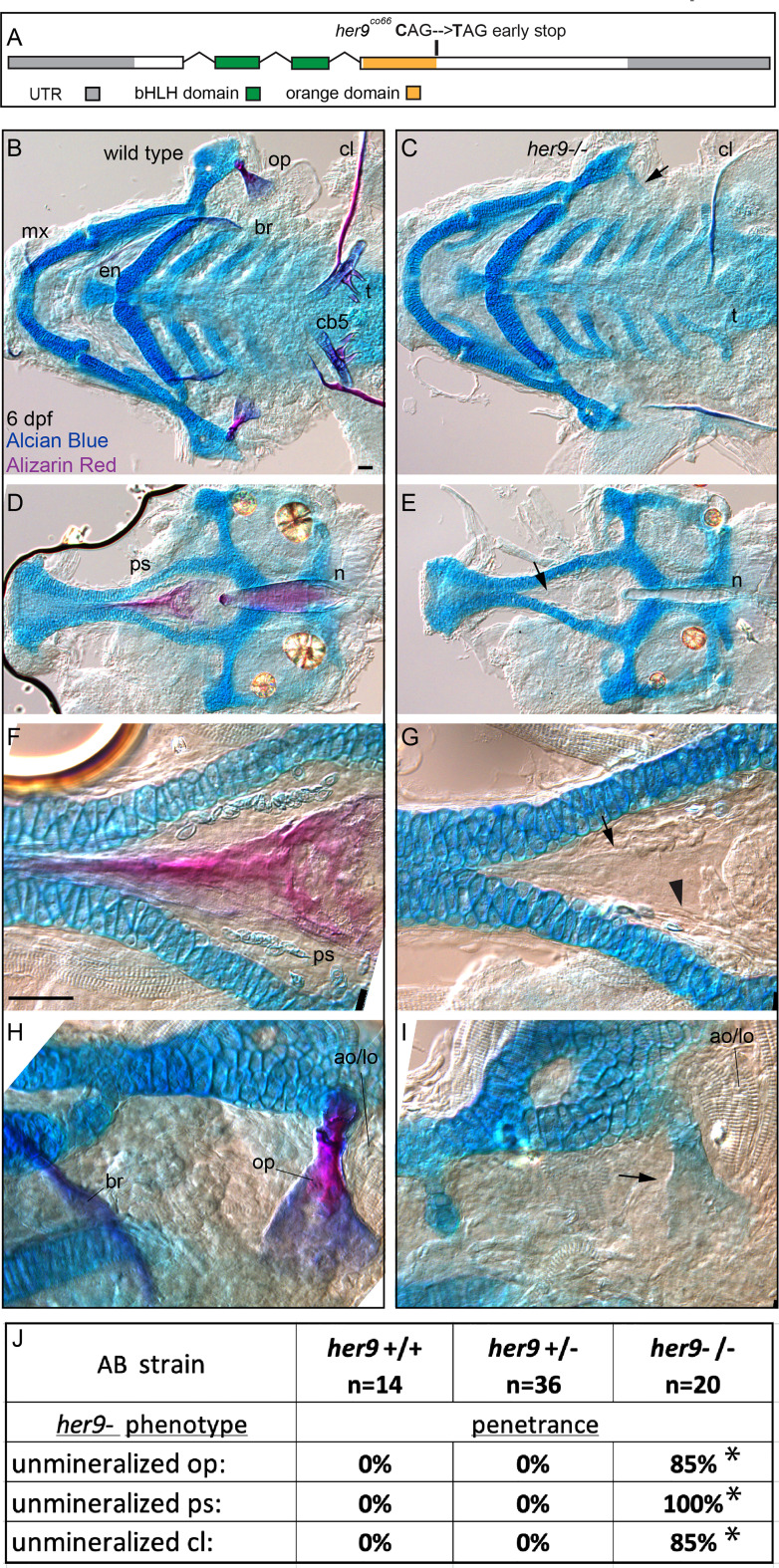
*her9* is required for efficient mineralization. **(A)** Exon diagram for *her9* indicating the untranslated regions (UTR) and the regions predicted to encode the basic helix-loop-helix (bHLH) and Orange domains. The location of the early stop encoded by the *her9^co66^* allele is indicated. **(B, C)** Zebrafish heterozygous for *her9* were pairwise intercrossed and six days post fertilization (dpf) larvae were stained with Alcian Blue and Alizarin Red to label cartilage and bone. The individuals were then genotyped, the viscerocrania were dissected, flat mounted, and then imaged. The following craniofacial skeletal elements are indicated in the wild-type individual: opercle bone (op), branchiostegal ray (br), maxilla (mx), entopterygoid (en), ceratobranchial 5 (cb5) bone, teeth (t), and the cleithrum (cl). Arrow in C indicates the unmineralized, Alcian Blue-positive opercle. Scale bar is 50 μm **(D, E)** The neurocrania from the same experiments as B and C were dissected, flat mounted, and then imaged. The parasphenoid bone (ps) and notochord (n) is indicated in the wild-type individual. Arrow in D indicates the shadow bone where the mineralized parasphenoid would be observable in wild types. **(F, G)** Enlargements from D and E. Arrow indicates the unmineralized shadow bone. Arrowhead denotes osteoblast-shaped cells surrounding the shadow bone. Scale bar is 50 μm **(H, I)** Enlargements from **(B, C)**. The adductor operculi and the levetor operculi muscles (ao/lo) are indicated. Arrow in I indicates the unmineralized, Alcian Blue-positive opercle with the adductor operculi and the levetor operculi muscles (ao/lo) attached. **(J)** The penetrance of *her9* mutant-associated phenotypes observed in 6 dpf larvae for each genotype are indicated. Asterisk indicates significant difference (p<0.05) in penetrance between the indicated genotype and wild type by Fishers exact test.

The *jag1b*-dependent *her6*;*her9* expression predicts that loss of function mutations in these genes will produce similar phenotypes to *jag1b* mutants. To test this prediction, we first examined *her9* individually. We recovered a *her9* mutant allele from an ENU mutagenesis screen (see Methods for details). The mutant allele produces a premature termination codon at amino acid 148 of 291, near the end of the region predicted to encode the Orange domain (**Figure 2A**). We detected reduced *her9* transcript levels in homozygous mutants suggesting that this lesion impacts *her9* function (**Figure S3**). When we crossed adults heterozygous for this *her9* mutation we failed to recover homozygous mutants from these crosses at 12 dpf, indicating that the mutation is homozygous lethal, as reported previously (22). We next stained offspring from these crosses with Alcian Blue and Alizarin Red at 6 dpf. Alcian Blue labels cartilage by interacting with mucopolysaccharides (30). Alizarin Red stains bone by complexing with calcium in mineralized bone (31). We observed that cartilage staining in *her9* mutants was overtly normal (**Figures 2B–I**), although the overall size of the craniofacial skeleton, including the cartilage elements, was significantly smaller than wild type-controls (**Figure S4**). In contrast to the cartilage skeleton, which is strongly stained with Alcian Blue, we observed dramatic reductions in Alizarin Red-stained bone in these mutants. We found reduced mineralization in cranial neural crest cell-derived structures like the opercle bone, branchiostegal rays, maxilla, entopterygoid, ceratobranchial 5 (cb5) bones and teeth. The cleithrum, which is derived from mesoderm (32), was also weakly and variably stained in *her9* homozygous mutants (**Figure 2C**). We also observed that notochord mineralization was defective in *her9* mutants (**Figures 2D, E**). Thus, *her9* functions in the mineralization of structures derived from both cranial neural crest cells as well as mesoderm. Scoring the individual bones for mineralization defect penetrance revealed that parasphenoid mineralization loss is 100% penetrant, while the cleithrum and the opercle mineralization losses are more variable (**Figure 2J**). Thus, all bones require *her9* function, but some boney structures are more sensitive to *her9* loss than others. Both scRNA-seq and *in situ* gene expression studies indicate that *her9* is broadly expressed during the pharyngula period (**Figures 1A**,**S3**), consistent with the broad defects in bone mineralization.

**Figure 3:**
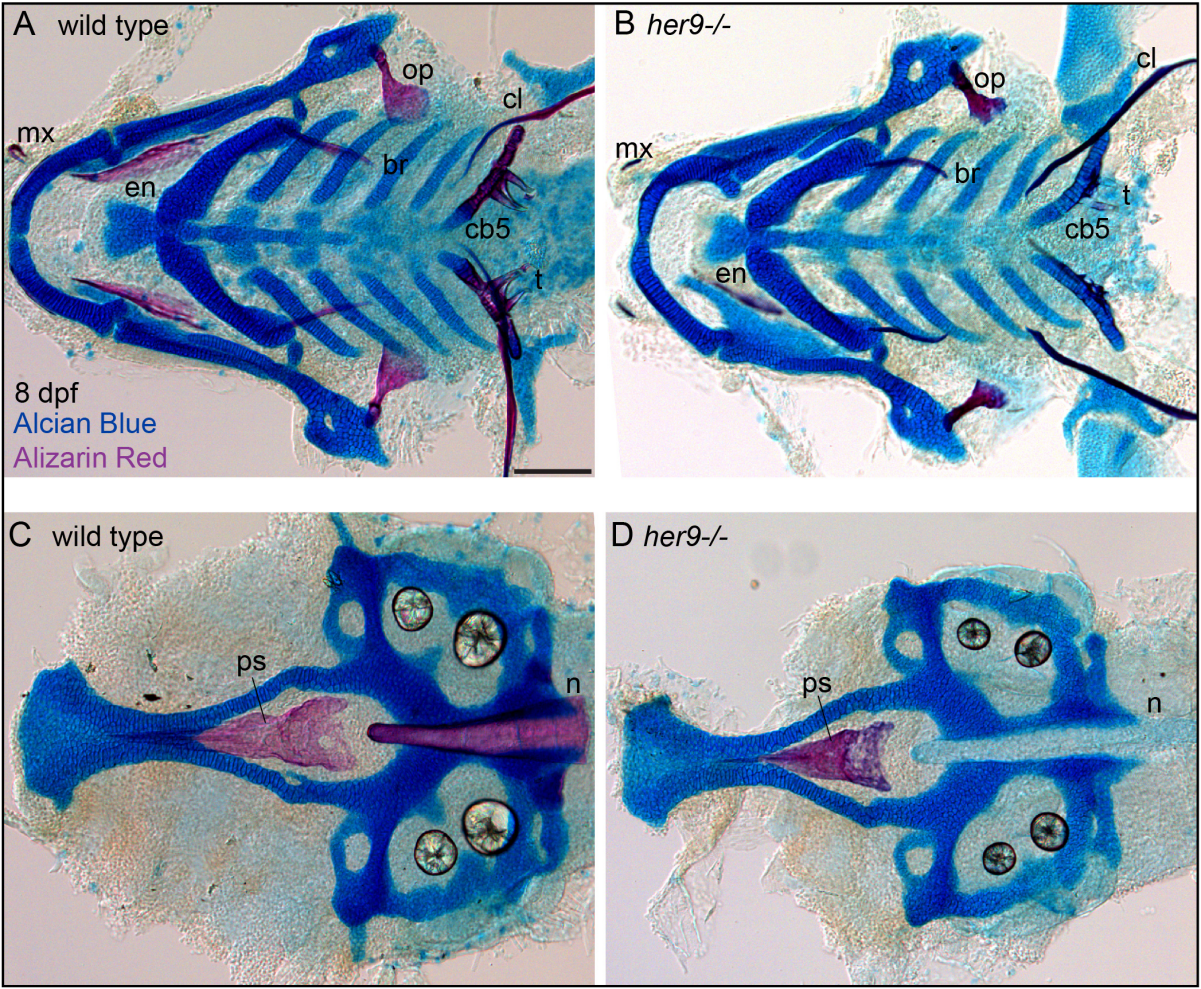
Mineralization defects in *her9* homozygous mutants partially recover at late stages of larval development. Zebrafish heterozygous for *her9* were pairwise intercrossed and at eight days post fertilization (dpf) larvae were stained with Alcian Blue and Alizarin Red to label cartilage and bone. The individuals were then genotyped, the viscerocrania and neurocrania were dissected, flat mounted, and then imaged. **(A, B)** Viscerocrania from wild-type and her9 homozygous mutant larvae. **(C, D)** Neurocrania from wild-type and her9 homozygous mutant larvae. The following craniofacial elements are indicated: opercle bone (op), branchiostegal ray (br), maxilla (mx), entopterygoid (en), ceratobranchial 5 (cb5) bone, teeth (t), cleithrum (cl), parasphenoid bone (ps), and notochord (n). Scale bar is 100 μm.

We next aimed to determine if mineralization phenotype in *her9* mutants persists into later stages of skeletal development. To this end, we analyzed *her9* mutant and wild-type sibling control skeletons in 8 dpf zebrafish (**Figure 3**). Strikingly we observed that while bone mineralization was still reduced in *her9* mutants, some mineralization had occurred. Specifically, the opercle and parasphenoid had reduced, but clearly observable, Alizarin Red staining at this stage compared with wild-type controls. Thus, the early strong mineralization defects in *her9* mutants can partially recover at later stages of skeletal development.

To determine if this bone mineralization phenotype was due to *her9* function downstream of Notch signaling, we treated wild-type embryos with the pharmacological gamma secretase inhibitor DBZ, which inhibits Notch intramembranous cleavage and release of NICD, and phenocopies the *jag1b* mutant (13, 15). When we treated embryos during the craniofacial patterning stage (18-48 hpf), DBZ produced craniofacial phenotypes reminiscent of the *jag1b* mutant phenotype (**Figure S5**). Like *jag1b* mutants, there was no detectable bone mineralization phenotype in these treated animals. When we treated later stage animals during chondrocyte and osteoblast differentiation (48-72 hpf) with DBZ, there were no overt changes to the craniofacial skeleton. However, this treatment window produced a body-curvature phenotype indicating that DBZ is affecting Notch signaling outside the head during this stage. Finally, when we treated developing fish at later stages while the skeleton grows and becomes more elaborate (72-96 hpf) with DBZ, they were indistinguishable from untreated controls. Longer treatments from 18-72 hpf produce dramatic global defects and cannot be meaningfully interpreted for mineralization. These results indicate that inhibiting Notch cleavage and NICD release does not phenocopy the *her9* phenotype but does phenocopy the *jag1b* phenotype. Thus, the role that *her9* plays in bone mineralization is likely independent of Notch cleavage and *jag1b* function.

There was only weak or no Alizarin Red staining in 6 dpf *her9* mutant craniofacial bony elements. However, when we closely examined these preps, we observed ‘shadow bones’ or vacant spaces shaped like the wild type structures where the elements would normally develop in wild types. For some of these, like the opercle, there was Alcian Blue staining of the unmineralized element (**Figure 2C**). Interestingly, the adductor operculi and levator operculi muscle groups which articulate with the posterior edge of the opercle at this stage appeared to be present and articulated with the unmineralized opercle. For some structures like the parasphenoid, there was no detectable Alcian Blue, just a shadow bone in the shape of the wild-type bone observable by Nomarski imaging (**Figure 2E**). High magnification images of the shadow bones revealed osteoblast-shaped cells in the expected position surrounding the element (**Figure 2G**). These findings motivate the hypothesis that the cells which normally produce the affected bony elements in wild types, may be present in the correct location but are variably unable to produce mineralized matrix with the correct timing.

### *her9* is not required for osteoblast transcriptional identity

**Figure 4:**
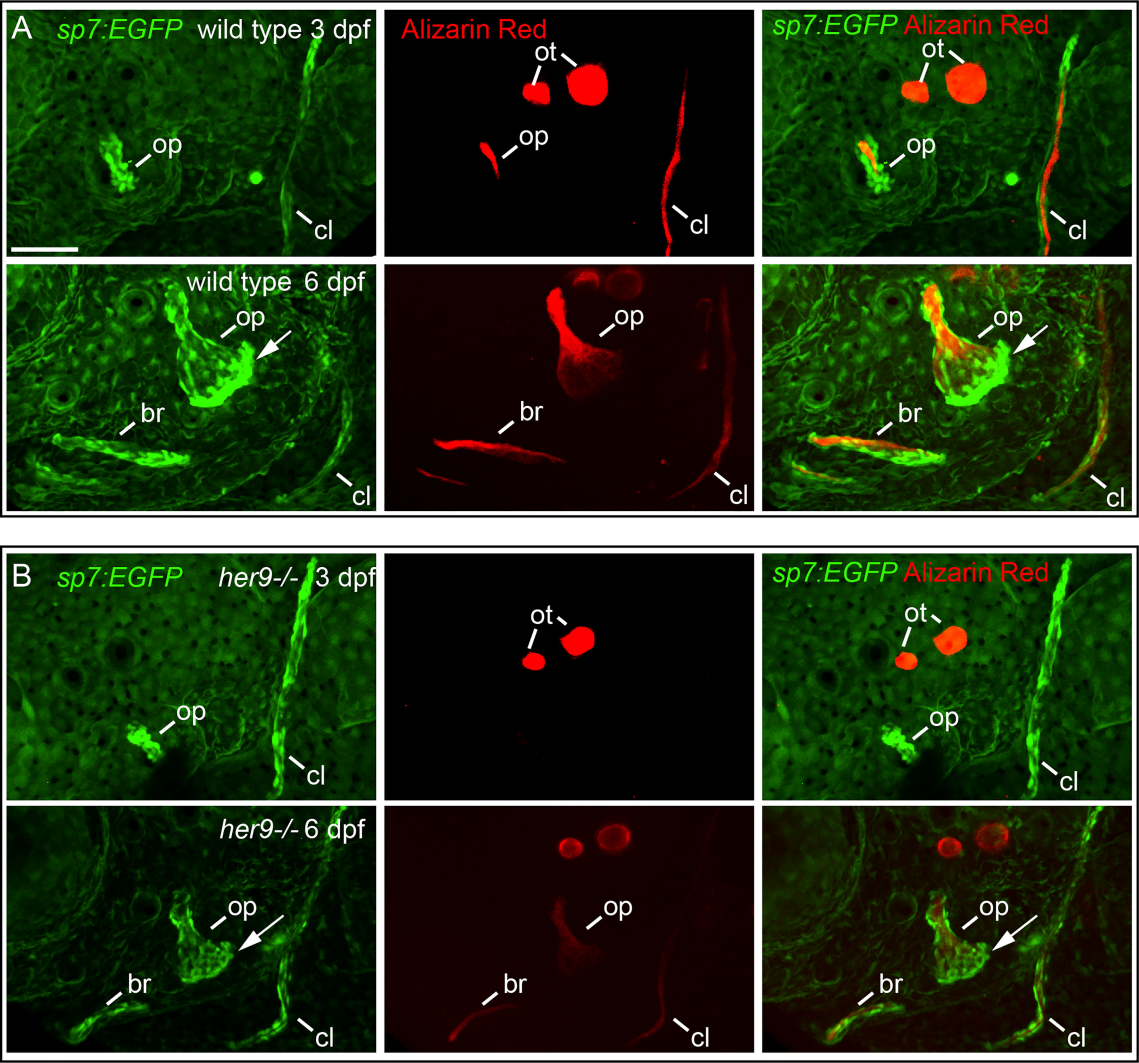
Osteoblasts differentiate in a spatiotemporal pattern similar to wild types in *her9* mutants, but only weakly mineralize bone. *sp7:EGFP*;*her9* heterozygotes were crossed to *her9* heterozygotes and offspring were sorted for transgene expression. 16 live transgenic animals were labeled with Alizarin Red and imaged at 3 dpf. After imaging, animals were recovered and grown in individual wells until 6 dpf when they were labeled with Alizarin Red and imaged again. Individual animals were recovered for genotyping to identify homozygous wild types **(A)** and homozygous *her9* mutants **(B)** that were imaged twice. The following structures are indicated: opercle bone (op), branchiostegal ray (br), cleithrum (cl), otoliths (ot). Arrows indicate osteoblasts prominently detected along the ventral edge of the opercle at 6 dpf. Scale bar is 50 μm.

**Figure 5:**
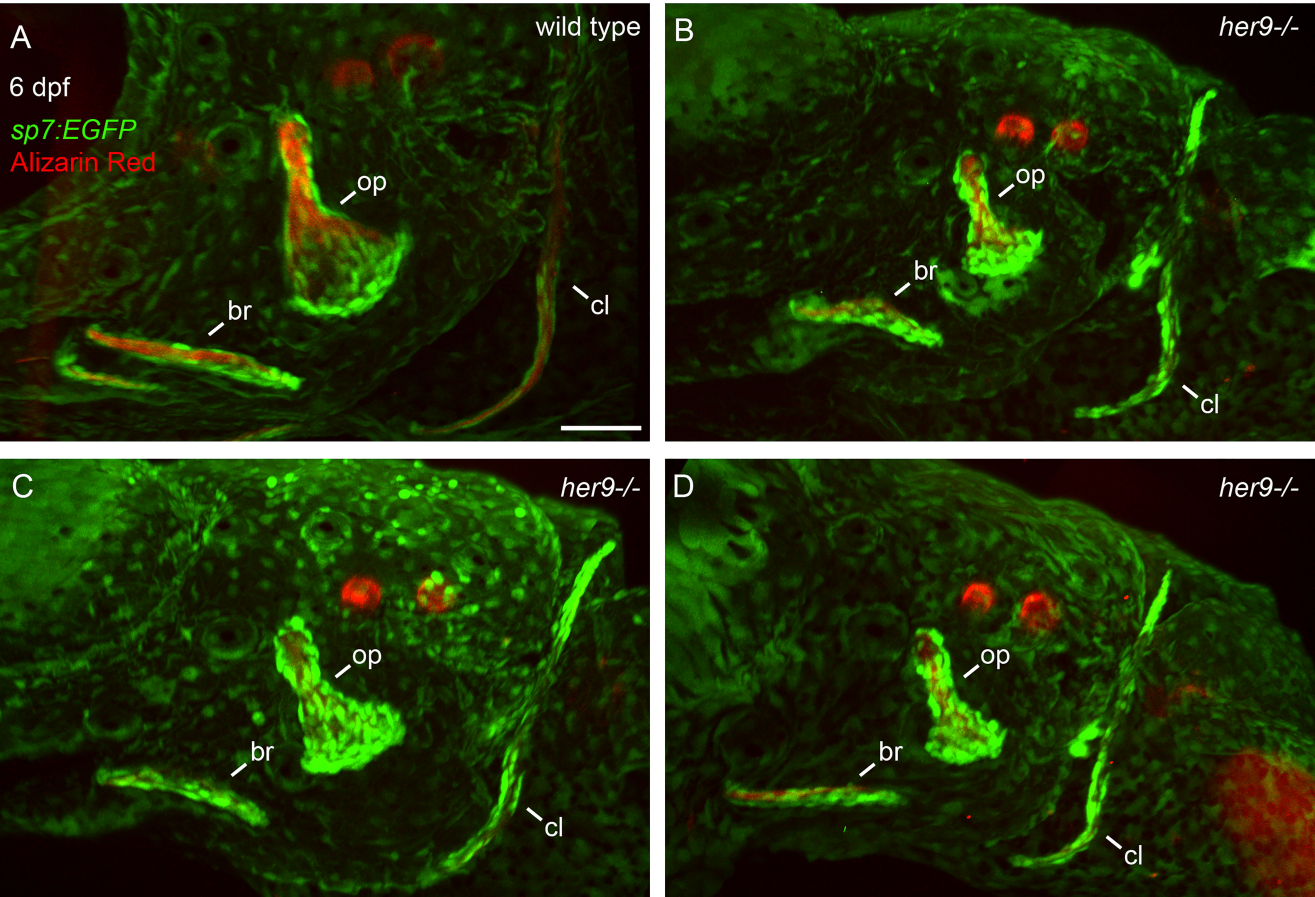
Opercle shapes are variable in *her9* mutants. *sp7:EGFP*;*her9* heterozygotes were crossed to *her9* heterozygotes and offspring were sorted for transgene expression. 24 live transgenic animals were labeled with Alizarin Red and imaged at 6 dpf. Imaged animals were genotyped. **(A)** Representative opercle region from a wild-type larva. **(B–D)** Three different her9 mutant larvae are shown. The following structures are indicated: opercle bone (op), branchiostegal ray (br), cleithrum (cl). Scale bar is 50 μm.

To determine whether the cells we observed surrounding the shadow bones are differentiated osteoblasts, we crossed the *her9* mutant into the *sp7:EGFP* transgenic line which faithfully labels active, bone-secreting osteoblasts (33). Fish with this transgene can be assayed with live Alizarin Red staining, which is a highly sensitive method of labeling mineralized bone (34). A strength of this approach is that the same bone can be longitudinally tracked in an individual over developmental time. We first examined the developing opercle at 3 dpf, when the initial opercle osteoblasts appear (35). In wild types, the first osteoblasts outline the early spicule-shaped opercle bone (**Figure 4A**) (36). By 6 dpf, the majority of osteoblasts are closely associated with the rapidly growing ventral edge. Examining the cells and bone matrix together at 6 dpf reveals that the early-forming spicule is the most mineralized and has the fewest osteoblasts, while the rapidly growing ventral edge, with the most osteoblasts, is more weakly mineralized. Similarly, the older, anterior end of the branchiostegal ray has fewer osteoblasts and more mineralized matrix, while the newly forming posterior end has the most osteoblasts and the least mineralized matrix. These observations indicate that we are tracking live, local mineralized matrix deposition by transgenic osteoblasts in areas of rapidly growing bone. In *her9* mutants, the earliest opercle osteoblasts are observable in a similar pattern to wild type, outlining a stick shape (**Figure 4B**). These results support our hypothesis that the cells surrounding the shadow bones (**Figure 2**) are indeed osteoblasts. Like wild types, by 6 dpf most of the *her9* mutant osteoblasts are located along the growing ventral edge. However, in contrast to wild types, the space outlined by osteoblasts is only very weakly stained with Alizarin Red at both 3 and 6 dpf (**Figure 4B**). Of note, the live fluorescent method of Alizarin Red staining was able to detect weakly mineralized matrix in *her9* mutants which was not observable in fixed skeletal preps imaged with transmitted light (**Figure 2**). Previous reports also indicate that Alizarin Red fluoresecence can reveal mineralized structures that are difficult to observe with visible light (37). This discovery that there was some mineralized bone in homozygous mutants are consistent with our late-stage study above (**Figure 3**) demonstrating that mineralized structures can be found in *her9* mutants at 8 dpf. Thus, mineralization is not completely absent in these mutants. Nevertheless, the amount of mineralized bone we are able to detect by both measures, at all stages, is severely reduced in *her9* mutants. In contrast to the bone, the otoliths are strongly stained with Alizarin red in both wild types and mutants, serving as an internal staining control and demonstrating that otolith mineralization is not dependent upon *her9* function. However, the otoliths are smaller in *her9* mutants compared with wild-type controls. Like other zebrafish craniofacial mutants (15, 35, 38), there is variation among mutant phenotypes; the shape of the opercle varies among *her9* mutant individuals (**Figure 5**). In spite of this variation, the *her9* mutant opercle bones are consistently smaller than wild-type opercles. Together with our cartilage measurements described above (**Figure S4**), we conclude that the entire craniofacial complex is smaller in *her9* mutants compared with wild types, and that *her9* mutants produce osteoblasts, which appear with the same spatiotemporal dynamics as wild types, but *her9* mutant osteoblasts are not able to secrete mineralized matrix with the same spatiotemporal dynamics.

### *her9* expression is not enriched in osteoblasts

**Figure 6:**
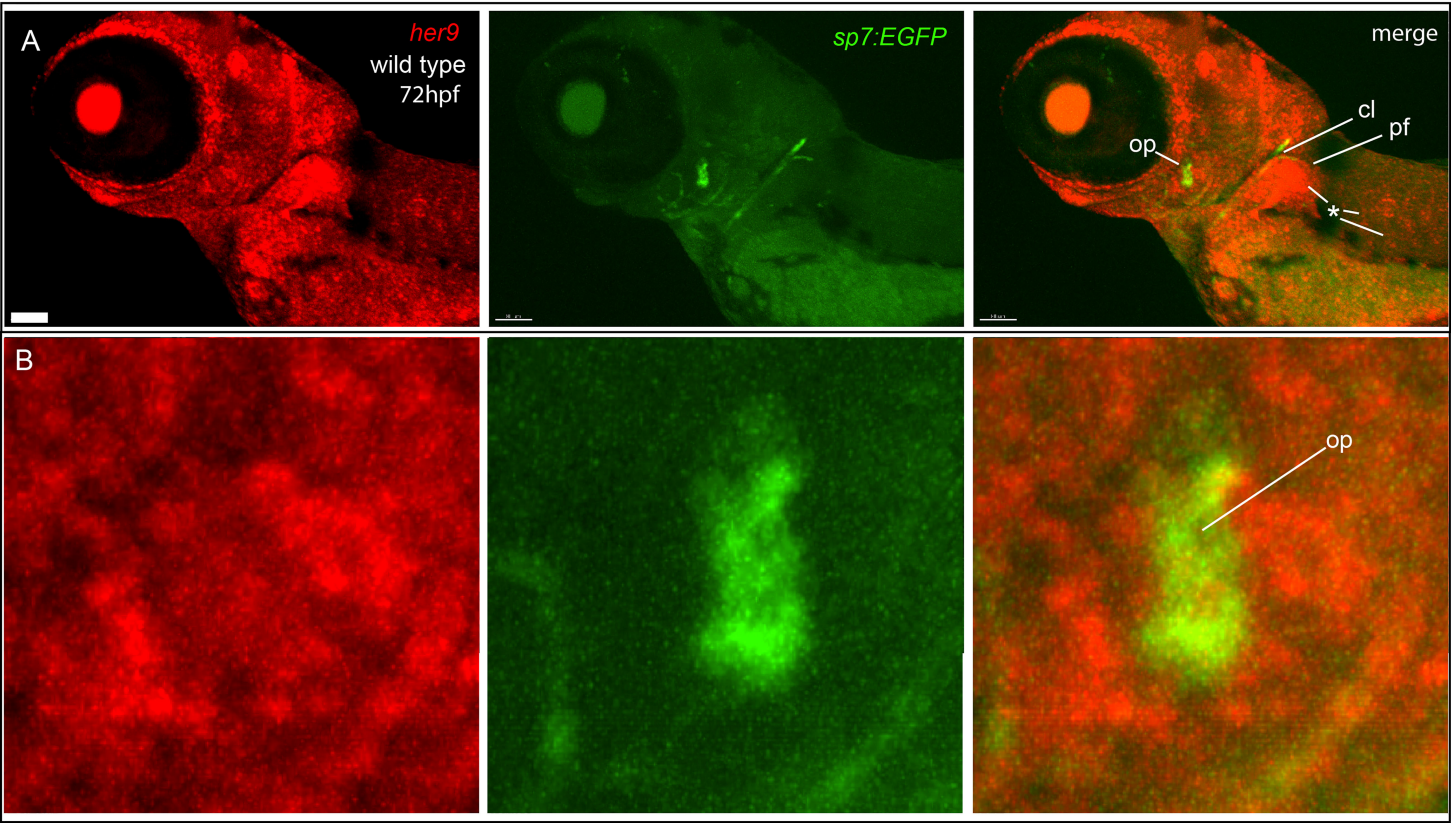
*her9* is broadly expressed, but not enriched in osteoblasts. **(A)** *In situ* hybridization detects broad *her9* expression at 72 hpf. Asterisks indicate areas of strong *her9* expression. Opercle osteoblasts do not strongly express *her9*. **(B)** Enlargements of images in **(A)**. Images in **(B)** are enlargements of **(A)**. Scale bar is 80 μm.

*her9* function is required for fish to efficiently mineralize bone. Therefore, we hypothesized that *her9* would be strongly expressed in osteoblasts. We tested this hypothesis by performing *in situ* hybridization in the *sp7:EGFP* transgenic background to monitor *her9* expression while labeling osteoblasts. We found that *her9* is broadly expressed at 72 hpf, but expression was not concentrated in osteoblasts that are actively secreting matrix (**Figure 6**). Thus, while *her9* expression at this stage was detected in the pectoral fin, and in the trunk, surprisingly *her9* was not strongly expressed in the osteoblasts themselves.

### *her9* genetically interacts with *jag1b* during craniofacial patterning

**Figure 7:**
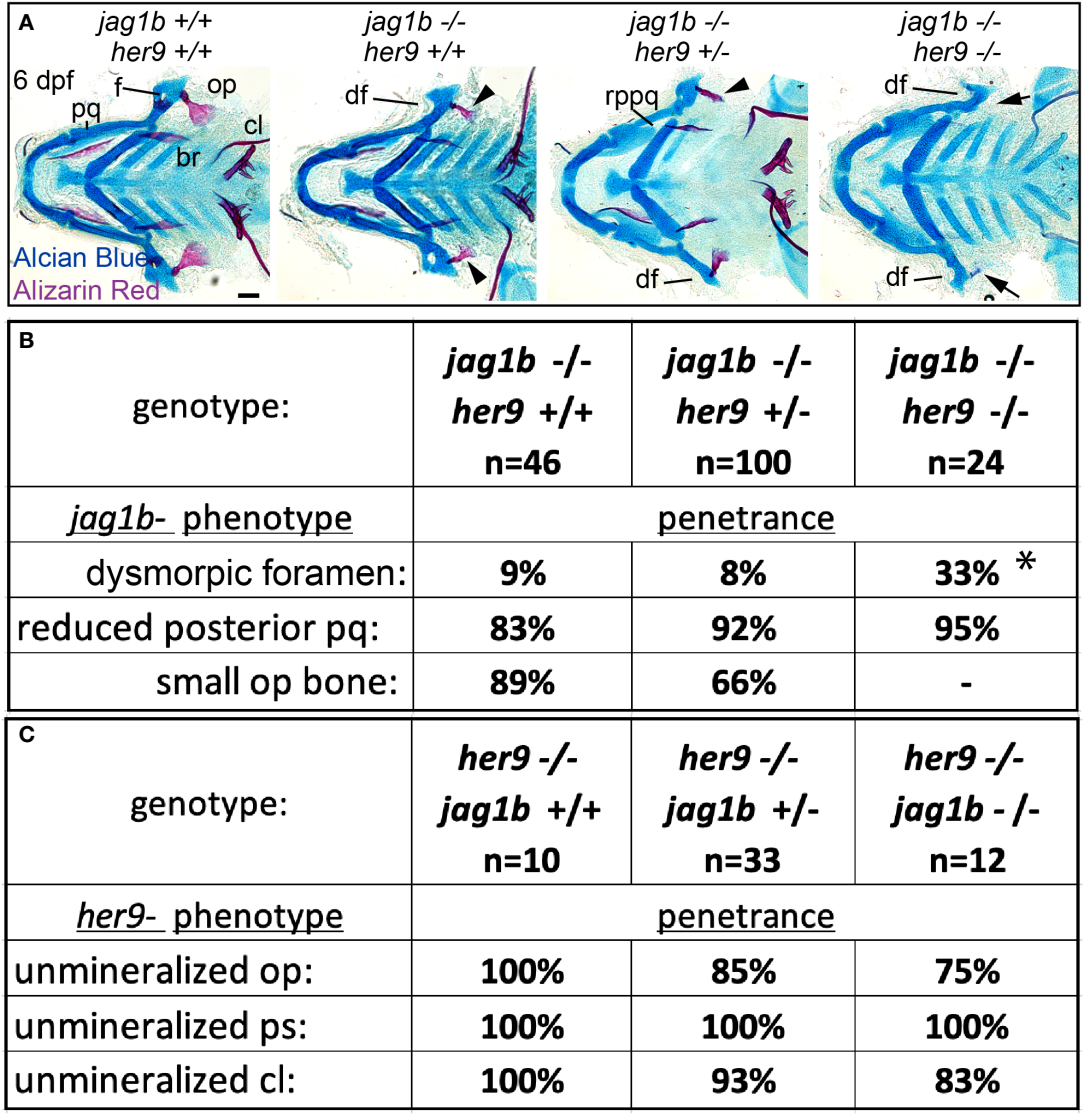
Mutations in *her9* enhance *jag1b* mutant phenotypes. **(A)** *jag1b*;*her9* double heterozygotes were intercrossed and six days post fertilization (dpf) larvae were stained with Alcian Blue and Alizarin Red to label cartilage and bone. The individuals were then genotyped, the viscerocrania were dissected and flat mounted then imaged. The following craniofacial skeletal elements are indicated in the wild-type individual: opercle bone (op), branchiostegal ray (br), cleithrum (cl), foramen (f) palatoquadrate (pq). The dysmorphic foramen (df) and reduced posterior pq (rppq) phenotypes are indicated, and the small op bone phenotype is indicated by an arrowhead. Arrows mark the opercle mineralization defect associated with *her9* homozygous mutants. Poorly stained posterior arch derived cartilages and one missing entopterygoid bone in *jag1b*-/-;*her9*+/- are staining and mounting artifacts in this individual and not representative phenotypes associated with this genotype. Scale bar is 50 μm **(B)** Genotyped preps from A were scored for penetrance of *jag1b* mutant-associated phenotypes. Asterisk in dysmorphic foramen row indicates that *jag1b*-/-;*her9*-/- is significantly different from both *jag1b*-/-;*her9*+/+ and *jag1b*-/-;*her9*+/- using a Fisher’s exact test with p<0.05. **(C)** Genotyped preps from A were scored for penetrance of *her9* mutant-associated phenotypes.

By labeling osteoblasts and weakly mineralized bone with *sp7:EGFP* and live Alizarin Red staining, respectively, we are able to observe opercle shape even when mineralization defects are severe in *her9* mutants (**Figures 4, 5**). Using this protocol, we observed divergence from the characteristic fan-shaped opercle in some individuals. We discovered that, occasionally, *her9* mutant opercles at 6 dpf were less fan shaped, and instead were more stick-like, reminiscent of *jag1b* mutants (**Figure 5**). Similarly, the opercle bone was more stick shaped than wild-type controls at 8 dpf. These observations motivated the hypothesis that in addition to a Notch-independent role in osteoblast mineralization, *her9* may also function to pattern the craniofacial skeleton downstream of *jag1b*. To test this hypothesis, we performed a genetic interaction experiment between *jag1b* and *her9*. We intercrossed *jag1b*;*her9* double heterozygotes and stained offspring of these crosses with Alcian Blue and Alizarin red (**Figure 7A**). By scoring phenotypes in genotyped larvae at 6dpf, we discovered *her9* and *jag1b* genetically interact. Removing both copies of *her9* from *jag1b* homozygous mutants significantly increased the penetrance of an “dysmorphic foramen” phenotype in the dorsal hyomandibular cartilage (**Figure 7B**). This dysmorphic foramen class includes mildly affected animals with a larger foramen as well as more severe individuals no identifiable foramen. These more severe phenotypes were previously interpreted to be due to the dorsal hyomandibular cartilage transforming to be more rod shaped, partially resembling the ventral ceratohyal cartilage (1). However, we did not observe any significant changes in the penetrance of the dorsal arch 1 phenotype, a reduced palatoquadrate. We cannot meaningfully score double mutants for opercle bone phenotypes because the bone is not labeled with alizarin red in these fixed preps. We did not observe any changes in the penetrance of the *her9*-associated mineralization phenotype when *jag1b* was removed. These results indicate that *her9* and *jag1b* genetically interact during patterning of the dorsal hyomandibular skeleton, but not during bone mineralization.

### *her6* genetically interacts with *jag1b* and *her9*

**Figure 8:**
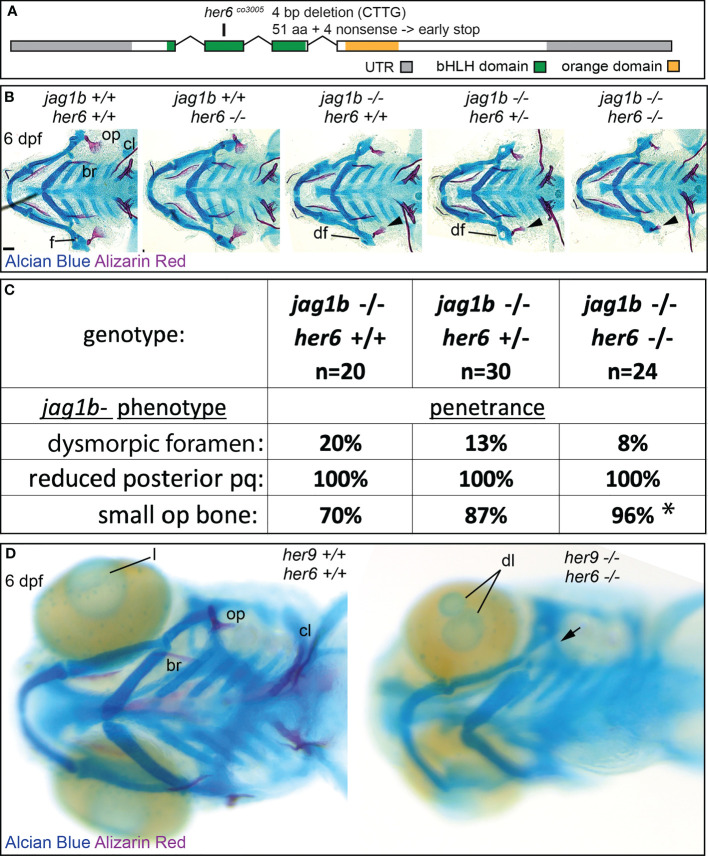
Mutations in *her6* enhance the *jag1b* mutant phenotype, *her6* and *her9* are redundantly required for eye development. **(A)** Exon diagram for *her6* indicating the untranslated regions (UTR) and the regions predicted to encode the basic helix-loop-helix (bHLH) and Orange domains. The location of the deletion encoded by the *her6^co3005^* allele is indicated. **(B)** *jag1b*;*her6* double heterozygotes were intercrossed and six days post fertilization (dpf) larvae were stained with Alcian Blue and Alizarin Red to label cartilage and bone. The individuals were then genotyped, the viscerocrania were dissected and flat mounted then imaged. The following craniofacial skeletal elements are indicated in the wild-type individual: opercle bone (op), branchiostegal ray (br), cleithrum (cl), foramen (f). The dysmorphic foramen phenotype (df) is indicated, and the small op bone phenotype is indicated by an arrowhead. Scale bar is 50 μm **(C)** Genotyped preps from B were scored for penetrance of *jag1b* mutant-associated phenotypes. Asterisk in small op bone row indicates that penetrance in *jag1b*-/-;*her6*-/- is significantly different from *jag1b*-/-;*her6*+/+ (p<0.05). **(D)** *her6*;*her9* double heterozygotes were intercrossed and six days post fertilization (dpf) larvae were stained with Alcian Blue and Alizarin Red to label cartilage and bone. The individuals were then genotyped and whole-mount imaged. An arrow indicates unmineralized opercle. The lens (l) is indicated in the wild type and a double lens phenotype is indicated in double homozygous mutants (dl).

Removing *her9* from *jag1b* mutants enhanced the *jag1b* craniofacial phenotype; double mutants had *jag1b* associated defects at higher penetrance than single mutants. However, penetrance was not increased to 100%. Therefore, we hypothesized that additional *her* genes might redundantly function downstream of *jag1b* during craniofacial patterning. *her6* is a known Notch target, and its cranial neural crest cell expression pattern makes it a strong candidate for functioning downstream of *jag1b* (**Figure 1**). A *her6* mutant was previously found to have a craniofacial skeleton indistinguishable from wild types (14). This mutant allele produces an eight-base pair deletion inducing a frameshift after amino acid 10 out of 270 then a premature termination codon after 12 missense amino acids. We speculated that this might not be a null allele, due to in-frame alternative start codons predicted in the transcript. Therefore, we generated a new mutation further downstream in *her6* that is a 4 base pair deletion causing a frameshift at amino acid 51, four nonsense amino acids, and an early stop at amino acid 55 (**Figure 8A**). Similar to the previously generated allele, our *her6* mutant also had a craniofacial skeleton that was indistinguishable from wild types (**Figure 8B**). We conclude that *her6* is not essential for craniofacial development in an otherwise wild-type background. Next, we tested whether the *her6* mutation genetically interacts with *jag1b*, like *her9* does. We crossed *jag1b*;*her6* double heterozygous mutants and stained offspring with Alcian Blue and Alizarin Red. When we scored genotyped, stained larvae, we found that no new phenotypes were produced. However, the penetrance of the *jag1b* mutant-associated reduced opercle bone was significantly increased bilaterally in *jag1b;her6* double homozygous mutants compared with *jag1b* single mutants (**Figures 8B, C**).

We next hypothesized that *her6* and *her9* might function redundantly during craniofacial patterning. To test this hypothesis, we combined the two mutations and intercrossed adults heterozygous for both *her6* and *her9*, and stained offspring with Alcian Blue and Alizarin Red (**Figure 8D**). Analyzing genotyped individuals revealed that the ceratohyal is occasionally misaligned at the midline in double homozygous mutants which is not observed in either single mutant. Further, fish double homozygous for both *her6* and *her9* developed eye phenotypes that were not present in either single mutant (**Figure 8D**). However, *her6*;*her9* double mutants did not phenocopy *jag1b* mutants, suggesting that other effectors provide further redundancy downstream of *jag1b.*

### *jag1b* and *her6* and *her9* together regulate dorsal versus ventral skeletal identity

**Figure 9:**
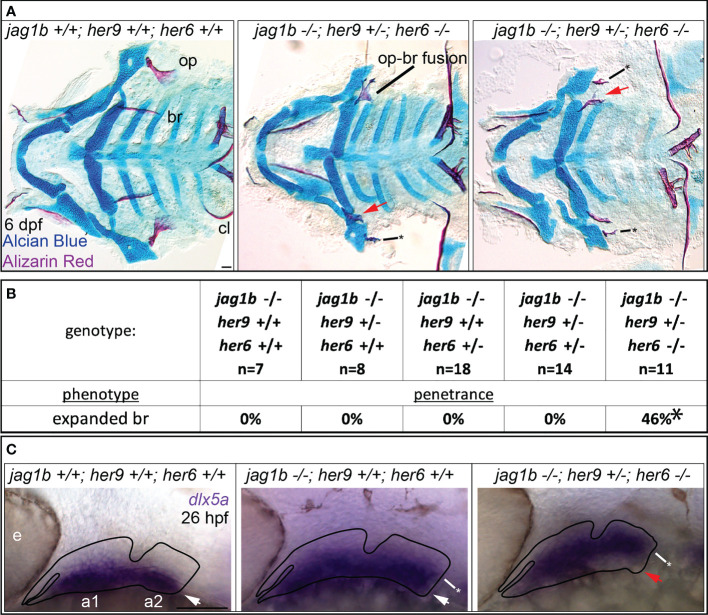
*jag1b*, *her9*, and *her6* redundantly regulate dorsal versus ventral identity. **(A)** *jag1b*;*her6;her9* triple heterozygotes were intercrossed and six days post fertilization (dpf) larvae were stained with Alcian Blue and Alizarin Red to label cartilage and bone. The individuals were then genotyped, the viscerocrania were dissected and flat mounted then imaged. The opercle (op), branchiostegal ray (br), and cleithrum (cl) are indicated in the wild type control. The bony fusion between the opercle and the branchiostegal ray is indicated. Red arrows indicate expanded, fan-shaped branchiostegal ray. Asterisk marks stick-shaped op phenotype **(B)** Genotyped preps from A were scored for penetrance of expanded branchiostegal ray. Asterisk indicates that penetrance of this phenotype in *jag1b*-/-;*her9*+/-;*her6*-/- is significantly different from all other genotypes, where it is never observed. **(C)** *jag1b*;*her6;her9* triple heterozygotes were intercrossed and 26 hours post fertilization (hpf) larvae were fixed for *in situ* hybridization experiments to label *dlx5a* expression. White arrows indicate ventral *dlx5a* expression, asterisks mark dorsally expanded *dlx5a* expression. Red arrow indicates reduced ventral *dlx5a* expression in triple mutants suggesting ventral-versus-dorsal identity reversal. This phenotype was observed on both sides of in 3/3 embryos examined with this genotype. Scale bars are 50 μm.

Our double mutant analyses demonstrated that *jag1b* genetically interacts with both *her9* and *her6*, and that *her9* and *her6* genetically interact with each other (**Figures 7**, **8**). While *her6* enhanced the *jag1b*-associated opercle phenotype, *her9* enhanced the *jag1b*-associated foramen phenotype. Based on these findings, we hypothesized that *jag1b* triple mutant combinations would produce novel craniofacial defects. Specifically, if *her* genes genetically interact with *jag1b* then dramatic phenotypes, more severe than the sum of the single mutant phenotypes, would be predicted in the triple mutants. When we intercrossed triple heterozygous fish and stained for Alcian Blue and Alizarin Red, we found that triple homozygous mutants were severely affected by general delay and edema producing secondary defects (**Figure S6**). This genotype could not be meaningfully scored for skeletal phenotypes. However, when we examined *jag1b* and *her6* homozygous mutants in combination with *her9* heterozygotes, we uncovered a new phenotype not seen in any other allelic combination in this study, the opercle and branchiostegal ray shapes were reversed (**Figure 9A**). Rather than a fan-shaped opercle and stick shaped branchiostegal ray, we observed a stick shaped opercle and a fan shaped branchiostegal ray. We found the branchiostegal ray fan phenotype in 46% of fish with this triple-mutant genotype, which was never present in any other genotype (**Figure 9B**). In some cases, the expanded branchiostegal ray was fused with the opercle bone forming a large plate-like structure (**Figure 9A**). These fusions were reminiscent of transformation phenotypes found when the Endothelin pathway is manipulated (39), motivating the hypothesis that these phenotypes are dorsoventral identity transformations. To directly test for molecular evidence of identity transformation, we performed *in situ* hybridization for the ventral arch gene *dlx5a* on these families (**Figure 9C**). Consistent with previous reports (1), we found that *dlx5a* expression was expanded dorsally in *jag1b* single mutants compared with wild-type controls. Strikingly, in the triple mutant genotype, we found that *dlx5a* expression was shifted dorsally. That is, *dlx5a* expression was present in the dorsal-most aspect of the second arch and absent ventrally in this arch.

## Discussion

### Nonredundant *her9* function in bone mineralization

We discovered that zebrafish *her9* is critical for timely mineralization. This mineralization role is nonredundant with other genes since loss of *her9* in an otherwise wild-type background produces fully penetrant mineralization defects, for at least some bony elements. We discovered that bones derived from neural crest cells, as well as those derived from mesoderm, both require *her9* function for mineralization. However, some bones (like the parasphenoid) never mineralize in *her9* mutants at 6 dpf. Meanwhile others (like the cleithrum) partially mineralize at this stage. These findings indicate that some bones are more sensitive to *her9* loss than others. We propose that the ancestral role of *her*/*hes* genes is in both patterning and mineralization, but that these functions were subfunctionalized resulting in *her9* primarily functioning in bone mineralization while retaining vestigial, partially redundant patterning function. In support, we recently proposed that vestigial, redundant paralog function can be retained and function to buffer against loss of one of the paralogs (40). It would be interesting to determine if there is a nonredundant patterning role of *her9* later in development. Some mineralization mutants display correct patterning of early-forming skeletal elements, but then develop late-stage defects in skull shape (41). However, we are unable to test for this possibility since the *her9* mutation is homozygous lethal. It is possible that generating new mutant *her9* alleles, or an allelic series, might produce different phenotypes and be informative for a more complete understanding of *her9* function.

It is interesting, and surprising, that *her9* expression is not enriched in osteoblasts. In *entpd5a* (*nob*) mutants, a mineralization phenotype similar to *her9* mutants develops (42). Unlike *her9* however, *entpd5a* is expressed in osteoblasts. Although expression of *entpd5a* in cells beyond osteoblasts is sufficient to rescue *entpd5a* mutants. Our data suggest that the mineralization role for *her9* is non cell-autonomous to the bone producing osteoblast cells. Determining the mechanism by which *her9* broadly controls mineralization, and any interaction with *entpd5a*, is a major focus for future study. We conclude that the *her9* cranial neural crest cell expression we detect by *in situ* and scRNAseq during early craniofacial development (24 hpf) functions in patterning downstream of *jag1b*, the mineralization function of *her9* is likely due to this gene functioning in other tissues, as broad expression is observed in *in situ* hybridization.

### *her9* and *her6* redundantly function in craniofacial patterning

Our study uncouples the mineralization and patterning roles of *her9.* In contrast to mineralization, the patterning role for *her9* is redundant with other family members since *her9* mutations do not affect patterning in an otherwise wild-type background, but do enhance patterning phenotypes when combined with other Notch pathway mutants. There are likely other Notch target genes, along with *her6* and *her9*, functioning in a patterning role downstream of *jag1b* since *her6*;*her9* double mutants do not phenocopy the *jag1b* phenotype. We found some evidence to suggest spatially distinct roles for different Notch target genes. Specifically, removing *her6* from *jag1b* mutants increases the penetrance of the small opercle bone phenotype bilaterally. Meanwhile removing *her9* from *jag1b* mutants increases penetrance of the foramen phenotype. Thus, different functions downstream of *jag1b* activation of Notch may have been subfunctionalized among *her*/*hes* genes. One caveat of this interpretation is that fixed skeletal preps from *her9* mutants cannot be scored for bone shape phenotypes, so we don’t know if homozygous *her9* mutants affect the penetrance of the stick opercle phenotype found in *jag1b* mutants. The *her* gene family is large, perhaps contributing to the extent of redundancy among family members. Future study disabling more *her* genes might reveal widespread redundancies during craniofacial development. Our previous work indicates that genes with stronger wild type expression are correlated with a larger buffering role when one family member is mutated (40). Therefore, our scRNA-seq dataset motivates examining *her8a* and *her11* next, since transcripts for these genes are detected, albeit weakly, in anterior arch cranial neural crest cells whereas many other family members are undetectable.

When *jag1b* and *her6* and *her9* function are disabled in combination, a branchiostegal ray phenotype develops that is not observed in any other combination. In this genotype, the branchiostegal ray was expanded into an opercle-like fan shape. At the same time, the opercle was reduced into a branchiostegal-like stick shape. These findings suggest both dorsal-to-ventral and ventral-to-dorsal homeotic transformations occur in this genotype. Consistently, we also found individuals with this genotype where the opercle and branchiostegal were fused, similar fusion phenotypes are observed in mutations in the *mef2ca* transcription factor encoding gene and are characterized as dorsoventral homeotic transformations (38, 43). Finally, we observed molecular evidence of homeotic transformation by examining *dlx5a* expression. *dlx5a* expression, which is ventrally restricted in wild types, is dorsally shifted in triple mutants. We interpret these data to indicate that dorsoventral identity is reversed in these triple mutants.

Mutations in *her6* do not produce an overt phenotype on their own but do genetically interact with other Notch pathway genes like *jag1b* and *her9*. Given this, it is curious that *her6* has been retained in the zebrafish genome. The *her*/*hes* genes can clearly be lost from genomes since the mouse ortholog of *her9*, *Hes4*, was lost. We propose that the *her*/*hes* family is highly redundant and interchangeable, and is therefore prone to gene losses and changes in functional requirement through evolutionary time.

The nonredundant and redundant roles of *her9* in mineralization and patterning, respectively, are also differentially dependent on Notch cleavage by gamma secretase. The mineralization role does not require Notch cleavage by gamma secretase since we are unable to phenocopy *her9* mutant phenotypes by inhibiting this enzyme. But this inhibitor does phenocopy the patterning phenotypes seen in *jag1b* mutants. Thus, it is possible that the mineralization role of *her9* is downstream of other signaling systems. For example, other reports indicate *her9* is in the Nodal and Retinoic Acid pathways (9, 22). This could be tested by inhibiting some of the other pathways associated with *her9* to test for phenocopy of the *her9* mineralization phenotype. Alternatively, *her9* may function downstream of gamma secretase-independent Notch activation (44). This alternative could be tested by mutating the relevant Notch receptors and ligands, or dominant-negative approaches to test for phenocopy of the mineralization phenotype, although previous studies utilizing these methods did not report a broad mineralization phenotype following Notch signaling blockade (16).

### Is the unmineralized bone in *her9* mutants osteoid bone?

Normally, bones form from the stepwise production of mineralized matrix. As osteoblasts mature, they first make osteoid bone, then mineralized matrix. We find that *her9* is required for the proper timing of the mineralization stages of bone formation. In *her9* mutants, osteoblasts differentiate in the correct position, with the normal timing, but they don’t mineralize the matrix with normal timing. However, there is detectible unmineralized matrix in some cases, like the opercle. Alcian Blue, which stains osteoid bone but not mature bone matrix (45), reproducibly labeled *her9* mutant opercles. Therefore, we interpret this *her9* mutant-associated phenotype as an extended osteoid stage. Intriguingly, extended osteoid stage opercles may occur naturally in some fish species, serving as a mechanism for generating bone shape diversity (46). In this work, the authors proposed that there are regions of extended osteoid in sculpin opercles contributing to opercle shape variation among different sculpin species. Thus, it is tempting to speculate that evolutionary changes in *her9* expression or function between different sculpin species underlie the extended osteoid observed in some sculpins. This would lead to naturally extended osteoid proposed to contribute to the diverse opercle bone shapes seen among sculpins. Further, that zebrafish *her9* mutants apparently produce an extended osteoid stage may inform human health. Rickets in children or osteomalacia in adults are disorders that arise when osteoid does not mineralize properly.

In this study we discovered a non-redundant, Notch-independent, bone mineralization requirement for *her9*. We also found a redundant role for *her9* and *her6* during Jag-Notch craniofacial patterning. These studies help us to understand human *HES4*-associated skeletal disease in an *in vivo* setting, which is not possible in rodent models. Our work helps to clarify the complex roles of *her*/*hes* genes during vertebrate skeletal development.

## Materials and methods

### Zebrafish strains and husbandry

All zebrafish were maintained and staged according to established protocols (47, 48). The *jag1b^b1105^* allele and genotyping protocol has been previously described (1). The transgenic line *Tg(sp7:EGFP)b1212* has been previously described (33).

The *her9^co66^* mutant line was generated by ENU mutagenesis and has a point mutation in exon 4 which produces an early stop codon near the predicted end of the Orange domain. The transition mutation converts the wild-type sequence GGGA**C**AGAT to GGGA**T**AGAT. The mutagenesis and mutant allele identification was performed as described (49). To identify the causative lesion, *co66* generated on the TU background was outcrossed to the AB strain for two generations and F3 embryos were screened. Heterozygous adults were intercrossed and 33 phenotypic wild type and 33 phenotypic mutant larvae were used to prepare pooled genomic DNA. Using 223 simple sequence-length polymorphism markers, we placed the *co66* locus on chromosome 23 between z7550, (31.6 centimorgan (cM), 13.6 megabase (mb)) and z7973 (34 cM, 26.0 mb). We identified *her9*, located at 23.4 mb as a candidate gene and found the mutation by Sanger sequencing which was homozygous only in phenotypically mutant individuals. The mutation was then maintained in our closed colony by outcrossing to AB for over seven generations, genotyping for *her9* heterozygotes at each generation. All individuals in this study were genotyped and the mineralization defect phenotype was 100% penetrant in *her9* homozygous mutants, and never present in heterozygotes or homozygous wild types. To genotype *her9^co66^*, a DNA fragment was amplified using primers Her9-FW-exon2, 5’-TCTTCAAAGCCAATCATGGAAA-3’, and Her9-RV-exon4, 5’-GAAGAGGCTGAGCCAAATGA-3’, and then digested with BsmFI to produce a 604 bp fragment in mutants and 539/66 bp fragments in wild types. We generated the *her6^co3005^* allele using CRISPR/Cas9 mutagenesis (50). We injected Cas9 mRNA along with two guide RNAs (5’- GCGAGAATCAACGAAAGCTT-3’ and 5’-CCGTTCTTGGGAAGTACCGA-3’) into single cell zebrafish embryos targeting exons 2 and 4 respectively. Injected zebrafish were screened for mutagenesis *via* PCR amplification using primers *her6* FW exon1 5’-GGCGAGCATGAACACTACA-3’ and *her6* RV exon4 5’-GGTTCCTGTCCACATGTGAA-3’. Sanger sequencing revealed a 4 bp deletion in exon 2 (AAAGGGTC) which is predicted to cause a frameshift after amino acid 10 of 270 and a premature stop codon after 12 missense amino acids. Sanger sequencing also showed a naturally occurring 5bp deletion in intron 2. Genotyping for *her6* was done using PCR amplification with primers *her6* geno FW 5’- GAGCATGAACACTACACCTGATA-3’ and *her6* geno RV 5’- CGAAAGTCCGACTGAGTCTTT-3’. The PCR product was then digested with HindIII to produce a 264bp fragment in mutants and 187/77 bp fragments in wild types. All zebrafish procedures have been approved by the University of Colorado Institutional Animal Care and Use Committee (IACUC), Protocol # 00188. Genomic DNA for genotyping was obtained by tail amputation. Fish were anesthetized by adding 0.01-0.2 mg/ml Tricaine (MS-222) to embryo media. Animals were euthanized by hypothermic shock followed by 1.5% sodium hypochlorite.

### Tissue staining

Alcian Blue and Alizarin Red were used to stain cartilage and bone, respectively as previously described (30, 51). Live Alizarin Red staining was performed on zebrafish as described (35) at 3 dpf and 6 dpf. Zebrafish larvae were placed in 0.01% Alizarin Red and 0.01% HEPES solution in E2 media and allowed to swim for 30 minutes. The zebrafish were then transferred to clean E2 media and imaged.

### Phenotype Scoring

Genotyped Alcian Blue and Alizarin Red stained zebrafish skeletons were scored for penetrance of relevant phenotypes. A Fisher’s Exact test was used to determine significant differences in penetrance between genotypes.

### Pharmacological Treatments

Notch cleavage inhibition using DBZ was performed similar to previously reported (13, 15). Wild-type zebrafish were treated with 10µM of DBZ or DMSO vehicle control from 18-48hpf, or 48-72hpf, or 72-96hpf. All treated zebrafish were raised to 6dpf and stained for bone and cartilage.

### RNA *in situ* hybridization

Whole mount *in situ* hybridization with fluorescence was performed as previously described (52). The *her9* and *dlx5a* probes have been previously described (9, 53).

### RT-qPCR

Gene expression studies were performed as previously described (15). Live individual 28 and 48 hpf embryos had their heads removed. Decapitated bodies were genotyped to identify homozygous wild-types and homozygous *jag1b* mutants. Heads from 5–6 identified homozygous wild types were pooled and total RNA was extracted with TRIzol Reagent from ThermoFischer Scientific. cDNA was prepared with Superscript III from Invitrogen. qPCR experiments utilized a Real-Time PCR StepOnePlus system from Applied Biosystems and SYBR green. A standard curve was generated from serially diluted (1:2:10) cDNA pools, and primers with a slope of -3.3 +- 0.3 were accepted. The relative quantity of target cDNA was calculated using Applied Biosystems StepOne V.2.0 software and the comparative Ct method. After surveying the expression of many housekeeping genes at multiple stages, we determined that *rps18* expression was the most consistent across stages and genotypes. Target gene expression in all experiments was normalized to *rps18*. Reactions were performed in technical triplicate, and the results represent two to six biological replicates. The following primers were used: *her9FW, 5’*-GAGAATCAACGAGAGCCTTG-3’, *her9REV* 5’-CTCCAG AATATCAGCTTTCTCC-3’, *her6FW 5’*-AACGAAAGCT TGGGTCAG-3’, *her6REV* 5’-ACTGTCATCTCCAGGATGT-3’, *rps18FW* 5’-CTGAACAGACAGAAGGACATAA-3’, *rps18REV* 5’-AGCCTCTCCAGATCTTCTC-3’.

### Cell sorting and sequencing

Cranial neural crest cells were sorted as described (29). Briefly, 60 wild-type 24 hpf *flia:EGFP*;*sox10:mRFP* double transgenic embryos were dissociated into single-cell suspension using cold-active protease from *Bacillus licheniformis*, DNase, EDTA, and trituration. Approximately 30,000 live, GFP/RFP double-positive cells were sorted into tubes pre-coated with fetal bovine serum with a MoFlo XDP100. Sorted cells at 2.08x10^6 cells/ml concentration with 70% viability were loaded into the 10x Chromium Controller aiming to capture 10000 cells. Using the NovaSEQ6000, 2×150 paired-end sequencing Chromium 10x Genomics libraries were sequenced at 100K reads per cell using 3prime NexGem V3.1(Dual Index) sequencing.

### scRNA-seq analysis

Recovered sequencing reads were mapped to a custom Cell Ranger reference based on the GRCz11/danRer11 genome assembly using the Cell Ranger pipeline (10X Genomics). The 10X Genomics Cell Ranger pipeline removed empty droplets from the dataset based on the distribution of cell barcodes. Additional analysis was performed in R using the Seurat package (54). Quality filtering of these data excluded cells with no genes detectable (>0 unique molecular identifiers (UMIs) per gene). Each gene in the dataset was required to be present in a minimum of five cells. Regarding the capture of dead or dying cells, a minimum presence of 250 genes (at <2.5% of mitochondrial UMIs detected) were required. After quality filtering, 7928 cells remained for analysis.

### Microscopy and image analysis

Alcian Blue and Alizarin Red skeletons were dissected, flat mounted, and then imaged on a Leica DMi8 inverted microscope equipped with a Leica DMC2900. All fluorescent imaging was performed on a Leica DMi8 equipped with an Andor Dragonfly 301 spinning disk confocal system. Live imaging was performed as previously described (55).

## Data availability statement

The sequencing dataset presented in this study can accessed from the GEO database (accession number GSE220081).

## Ethics statement

The animal study was reviewed and approved by University of Colorado IACUC.

## Author contributions

AS, AM-M, JS, MW and JMM designed performed and interpreted experiments and edited the manuscript. BA designed experiments. JN interpreted experiments wrote and edited the manuscript. All authors contributed to the article and approved the submitted version.

## References

[1] Zuniga E Stellabotte F Crump JG. Jagged-notch signaling ensures dorsal skeletal identity in the vertebrate face. Development. (2010) 137(11):1843–52. doi: 10.1242/dev.049056 PMC2867320 20431122

[2] Kraus JM Giovannone D Rydzik R Balsbaugh JL Moss IL Schwedler JL. Notch signaling enhances bone regeneration in the zebrafish mandible. Development (2022) 149(5):dev199995. doi: 10.1242/dev.199995 35178545 PMC8959151

[3] Pakvasa M Haravu P Boachie-Mensah M Jones A Coalson E Liao J. Notch signaling: its essential roles in bone and craniofacial development. Genes diseases. (2021) 8(1):8–24. doi: 10.1016/j.gendis.2020.04.006 33569510 PMC7859553

[4] Nichols JT Miyamoto A Weinmaster G. Notch signaling - constantly on the move. Traffic. (2007) 8(8):959–69. doi: 10.1111/j.1600-0854.2007.00592.x 17547700

[5] Artavanis-Tsakonas S Muskavitch MA. Notch: the past, the present, and the future. Curr topics Dev Biol (2010) 92:1–29. doi: 10.1016/S0070-2153(10)92001-2 20816391

[6] Kageyama R Ohtsuka T Kobayashi T. The Hes gene family: repressors and oscillators that orchestrate embryogenesis. Development (Cambridge, England) (2007) 134.7:1243–51 10.1242/dev.000786 17329370

[7] Kuretani A Yamamoto T Taira M Michiue T. Evolution of hes gene family in vertebrates: the hes5 cluster genes have specifically increased in frogs. Evol (2021) 21(1):1–15. doi: 10.1186/s12862-021-01879-6 PMC8320183 34325655

[8] Fisher AL Ohsako S Caudy M. The WRPW motif of the hairy-related basic helix-loop-helix repressor proteins acts as a 4-amino-acid transcription repression and protein-protein interaction domain. Mol Cell Biol (1996) 16(6):2670–7. doi: 10.1128/MCB.16.6.2670 PMC231257 8649374

[9] Latimer AJ Shin J Appel B. her9 promotes floor plate development in zebrafish. Dev dynamics: an Off Publ Am Assoc Anatomists. (2005) 232(4):1098–104. doi: 10.1002/dvdy.20264 15739223

[10] Radosevic M Robert-Moreno À Coolen M Bally-Cuif L Alsina B. Her9 represses neurogenic fate downstream of Tbx1 and retinoic acid signaling in the inner ear. Development. (2011) 138(3):397–408. doi: 10.1242/dev.056093 21205785

[11] Bae Y-K Shimizu T Hibi M. Patterning of proneuronal and inter-proneuronal domains by hairy-and enhancer of split-related genes in zebrafish neuroectoderm. Development (Cambridge, England) (2005) 132.6:1375–85. 10.1242/dev.01710 15716337

[12] Leve C Gajewski M Rohr KB Tautz D. Homologues of c-hairy1 (her9) and lunatic fringe in zebrafish are expressed in the developing central nervous system, but not in the presomitic mesoderm. Dev Genes evolution. (2001) 211(10):493–500. doi: 10.1007/s00427-001-0181-4 11702199

[13] Barske L Askary A Zuniga E Balczerski B Bump P Nichols JT. Competition between jagged-notch and Endothelin1 signaling selectively restricts cartilage formation in the zebrafish upper face. PloS Genet (2016) 12(4):e1005967. doi: 10.1371/journal.pgen.1005967 27058748 PMC4825933

[14] Askary A Xu P Barske L Bay M Bump P Balczerski B. Genome-wide analysis of facial skeletal regionalization in zebrafish. Development. (2017) 144(16):2994–3005. doi: 10.1242/dev.151712 28705894 PMC5592815

[15] Sucharov J Ray K Brooks EP Nichols JT. Selective breeding modifies mef2ca mutant incomplete penetrance by tuning the opposing notch pathway. PloS Genet (2019) 15(12):e1008507. doi: 10.1371/journal.pgen.1008507 31790396 PMC6907857

[16] Paudel S Gjorcheska S Bump P Barske L. Patterning of cartilaginous condensations in the developing facial skeleton. Dev Biol (2022) 486:44–55. doi: 10.1016/j.ydbio.2022.03.010 35358504 PMC9058241

[17] Oda T Elkahloun AG Pike BL Okajima K Krantz ID Genin A. Mutations in the human Jagged1 gene are responsible for alagille syndrome. Nat Genet (1997) 16(3):235–42. doi: 10.1038/ng0797-235 9207787

[18] Suzuki A Sangani DR Ansari A Iwata J. Molecular mechanisms of midfacial developmental defects. Dev Dynamics. (2016) 245(3):276–93. doi: 10.1002/dvdy.24368 PMC4755841 26562615

[19] Humphreys R Zheng W Prince LS Qu X Brown C Loomes K. Cranial neural crest ablation of Jagged1 recapitulates the craniofacial phenotype of alagille syndrome patients. Hum Mol Genet (2012) 21(6):1374–83. doi: 10.1093/hmg/ddr575 PMC3465692 22156581

[20] Akimoto M Kameda Y Arai Y Miura M Nishimaki T Takeda A. Hes1 is required for the development of craniofacial structures derived from ectomesenchymal neural crest cells. J Craniofacial Surgery. (2010) 21(5):1443–9. doi: 10.1097/SCS.0b013e3181ebd1a0 20818256

[21] Kameda Y Saitoh T Nemoto N Katoh T Iseki S Fujimura T. Hes1 is required for the development of pharyngeal organs and survival of neural crest-derived mesenchymal cells in pharyngeal arches. Cell Tissue Res (2013) 353(1):9–25. doi: 10.1007/s00441-013-1649-z 23686616

[22] Coomer CE Wilson SG Titialii-Torres KF Bills JD Krueger LA Petersen RA. Her9/Hes4 is required for retinal photoreceptor development, maintenance, and survival. Sci Rep (2020) 10(1):1–20. doi: 10.1038/s41598-020-68172-2 32647335 PMC7347560

[23] Shimada S Shimojima K Okamoto N Sangu N Hirasawa K Matsuo M. Microarray analysis of 50 patients reveals the critical chromosomal regions responsible for 1p36 deletion syndrome-related complications. Brain Dev (2015) 37(5):515–26. doi: 10.1016/j.braindev.2014.08.002 25172301

[24] Grogan SP Olee T Hiraoka K Lotz MK. Repression of chondrogenesis through binding of notch signaling proteins HES-1 and HEY-1 to n-box domains in the COL2A1 enhancer site. Arthritis Rheumatism. (2008) 58(9):2754–63. doi: 10.1002/art.23730 PMC2786215 18759300

[25] Dong Y Jesse AM Kohn A Gunnell LM Honjo T Zuscik MJ. RBPjκ-dependent notch signaling regulates mesenchymal progenitor cell proliferation and differentiation during skeletal development. Development. (2010) 137(9):1461–71. doi: 10.1242/dev.042911 PMC2853848 20335360

[26] Cakouros D Isenmann S Hemming SE Menicanin D Camp E Zannetinno ACW. Novel basic helix–loop–helix transcription factor Hes4 antagonizes the function of twist-1 to regulate lineage commitment of bone marrow stromal/stem cells. Stem Cells Dev (2015) 24(11):1297–308. doi: 10.1089/scd.2014.0471 25579220

[27] Hilton MJ Tu X Wu X Bai S Zhao H Kobayashi T. Notch signaling maintains bone marrow mesenchymal progenitors by suppressing osteoblast differentiation. Nat Med (2008) 14(3):306–14. doi: 10.1038/nm1716 PMC2740725 18297083

[28] Zhang Y Lian JB Stein JL Van Wijnen AJ Stein GS. The notch-responsive transcription factor hes-1 attenuates osteocalcin promoter activity in osteoblastic cells. J Cell Biochem (2009) 108(3):651–9. doi: 10.1002/jcb.22299 PMC3150580 19670267

[29] Mitchell JM Sucharov J Pulvino AT Brooks EP Gillen AE Nichols JT. The alx3 gene shapes the zebrafish neurocranium by regulating frontonasal neural crest cell differentiation timing. Development (2021) 148(7):dev197483. doi: 10.1242/dev.197483 33741714 PMC8077506

[30] Walker MB Kimmel CB. A two-color acid-free cartilage and bone stain for zebrafish larvae. Biotech Histochem (2007) 82(1):23–8. doi: 10.1080/10520290701333558 17510811

[31] Puchtler H Meloan SN Terry MS. On the history and mechanism of alizarin and alizarin red s stains for calcium. J Histochem Cytochemistry. (1969) 17(2):110–24. doi: 10.1177/17.2.110 4179464

[32] Kague E Gallagher M Burke S Parsons M Franz-Odendaal T Fisher S. Skeletogenic fate of zebrafish cranial and trunk neural crest. PloS One (2012) 7(11):e47394. doi: 10.1371/journal.pone.0047394 23155370 PMC3498280

[33] DeLaurier A Eames BF Blanco-Sanchez B Peng G He X Swartz ME. Zebrafish *sp7:EGFP*: a transgenic for studying otic vesicle formation, skeletogenesis, and bone regeneration. Genesis. (2010) 48(8):505–11. doi: 10.1002/dvg.20639 PMC2926247 20506187

[34] Eames BF DeLaurier A Ullmann B Huycke TR Nichols JT Dowd J. FishFace: interactive atlas of zebrafish craniofacial development at cellular resolution. BMC Dev Biol (2013) 13(1):23. doi: 10.1186/1471-213X-13-23 23714426 PMC3698193

[35] Nichols JT Blanco-Sanchez B Brooks EP Parthasarathy R Dowd J Subramanian A. Ligament versus bone cell identity in the zebrafish hyoid skeleton is regulated by mef2ca. Development. (2016) 143(23):4430–40. doi: 10.1242/dev.141036 PMC5201047 27789622

[36] Kimmel CB DeLaurier A Ullmann B Dowd J McFadden M. Modes of developmental outgrowth and shaping of a craniofacial bone in zebrafish. PloS One (2010) 5(3):e9475. doi: 10.1371/journal.pone.0009475 20221441 PMC2832765

[37] Bensimon-Brito A Cardeira J Dionísio G Huysseune A Cancela M Witten P. Revisiting *in vivo* staining with alizarin red s-a valuable approach to analyse zebrafish skeletal mineralization during development and regeneration. BMC Dev Biol (2016) 16(1):1–10. doi: 10.1186/s12861-016-0102-4 26787303 PMC4719692

[38] DeLaurier A Huycke TR Nichols JT Swartz ME Larsen A Walker C. Role of *mef2ca* in developmental buffering of the zebrafish larval hyoid dermal skeleton. Dev Biol (2014) 385(2):189–99. doi: 10.1016/j.ydbio.2013.11.016 PMC3892954 24269905

[39] Kimmel CB Walker MB Miller CT. Morphing the hyomandibular skeleton in development and evolution. J Exp Zool B Mol Dev Evol (2007) 308(5):609–24. doi: 10.1002/jez.b.21155 17358015

[40] Bailon-Zambrano R Sucharov J Mumme-Monheit A Murry M Stenzel A Pulvino AT. Variable paralog expression underlies phenotype variation. Elife. (2022) 11:e79247. doi: 10.7554/eLife.79247 36134886 PMC9555865

[41] Kague E Roy P Asselin G Hu G Simonet J Stanley A. Osterix/Sp7 limits cranial bone initiation sites and is required for formation of sutures. Dev Biol (2016) 413(2):160–72. doi: 10.1016/j.ydbio.2016.03.011 PMC5469377 26992365

[42] Huitema LF Apschner A Logister I Spoorendonk KM Bussmann J Hammond CL. Entpd5 is essential for skeletal mineralization and regulates phosphate homeostasis in zebrafish. Proc Natl Acad Sci (2012) 109(52):21372–7. doi: 10.1073/pnas.1214231110 PMC3535636 23236130

[43] Miller CT Swartz ME Khuu PA Walker MB Eberhart JK Kimmel CB. *mef2ca* is required in cranial neural crest to effect Endothelin1 signaling in zebrafish. Dev Biol (2007) 308(1):144–57. doi: 10.1016/j.ydbio.2007.05.018 PMC2148033 17574232

[44] Huppert SS Ilagan MXG De Strooper B Kopan R. Analysis of notch function in presomitic mesoderm suggests a γ-secretase-independent role for presenilins in somite differentiation. Dev Cell (2005) 8(5):677–88. doi: 10.1016/j.devcel.2005.02.019 15866159

[45] Baylink D Wergedal J Thompson E. Loss of proteinpolysaccharides at sites where bone mineralization is initiated. J Histochem Cytochemistry. (1972) 20(4):279–92. doi: 10.1177/20.4.279 4113296

[46] Cytrynbaum EG Small CM Kwon RY Hung B Kent D Yan YL. Developmental tuning of mineralization drives morphological diversity of gill cover bones in sculpins and their relatives. Evol letters. (2019) 3(4):374–91. doi: 10.1002/evl3.128 PMC6675512 31388447

[47] Westerfield M. The zebrafish book: A guide for the laboratory use of zebrafish (Brachydanio rerio). Eugene OR: University of Oregon Press (1993).

[48] Kimmel CB Ballard WW Kimmel SR Ullmann B Schilling TF. Stages of embryonic development of the zebrafish. Dev Dynamics. (1995) 203(3):253–310. doi: 10.1002/aja.1002030302 8589427

[49] Kearns CA Walker M Ravanelli AM Scott K Arzbecker MR Appel B. Zebrafish spinal cord oligodendrocyte formation requires boc function. Genetics (2021) 218(4):iyab082. doi: 10.1093/genetics/iyab082 34057474 PMC8864740

[50] Jao L-E Wente SR Chen W. Efficient multiplex biallelic zebrafish genome editing using a CRISPR nuclease system. Proc Natl Acad Sci (2013) 110(34):13904–9. doi: 10.1073/pnas.1308335110 PMC3752207 23918387

[51] Brooks E Nichols J. Shifting zebrafish lethal skeletal mutant penetrance by progeny testing. J visualized experiments: JoVE (2017) 127:p.e56200. doi: 10.3791/56200 PMC5614404 28892034

[52] Nichols JT Pan L Moens CB Kimmel CB. *barx1* represses joints and promotes cartilage in the craniofacial skeleton. Development. (2013) 140(13):2765–75. doi: 10.1242/dev.090639 PMC3678344 23698351

[53] Walker MB Miller CT Coffin Talbot J Stock DW Kimmel CB. Zebrafish *furin* mutants reveal intricacies in regulating Endothelin1 signaling in craniofacial patterning. Dev Biol (2006) 295(1):194–205. doi: 10.1016/j.ydbio.2006.03.028 16678149

[54] Butler A Hoffman P Smibert P Papalexi E Satija R. Integrating single-cell transcriptomic data across different conditions, technologies, and species. Nat Biotechnol (2018) 36(5):411–20. doi: 10.1038/nbt.4096 PMC6700744 29608179

[55] Sasaki MM Nichols JT Kimmel CB. edn1 and hand2 interact in early regulation of pharyngeal arch outgrowth during zebrafish development. PloS One (2013) 8(6):e67522. doi: 10.1371/journal.pone.0067522 23826316 PMC3691169

