# Supplementary material for: Distinct and redundant roles for zebrafish her genes during mineralization and craniofacial patterning
Source: Front Endocrinol (Lausanne). 2022 Dec 12;13:1033843. doi: 10.3389/fendo.2022.1033843 (PMC9791542; doi:10.3389/fendo.2022.1033843)
Supplement: Supplementary file 1 [file DataSheet_1.docx]

**SUPPLEMENTARY INFORMATION**

**SUPPLEMENTARY FIGURE 1: Many *her* genes are expressed in cranial neural crest cells, but *her6* and *her9* are the family members with the strongest expression in skeletogenic pharyngeal arch 1 and 2 cells.** UMAPs from the same experiment as Fig. 1 demonstrate expression of all *her* genes detectable in our scRNA-seq dataset.

**SUPPLEMENTARY FIGURE 2: *jag1b*, *her9*, and *her6* are coexpressed in the same population and occasionally in the same cells.** Coexpression plots for (A, B) *jag1b* and *her6*, (C, D) *jag1b* and *her9*, and (E, F) *her9* and *her6* demonstrate that these three genes are coexpressed in the same population of cranial neural crest cells. B, D, and F are enlargements of A, C, and E, respectively. Asterisks indicate examples of cells that co-express the indicated genes. (G) The anterior arch population was subsetted and reclustered. We include known arch markers in these analyses. *dlx2a* is a pan anterior arch marker, *dlx5a* is expressed in the ventral region of arches 1 and 2, and *hand2* is restricted to the ventral-most domain of arches 1 and 2. *jag1b*, *her9* and *her6* are all expressed in the dorsal domain of arches 1 and 2 which is *dlx2a* positive and *dlx5a* and *hand2* negative. Of note, *her6* and *her9* are not exclusive to the *jag1b* positive population.

**SUPPLEMENTARY FIGURE 3: *her9* is broadly expressed in cranial neural crest cells and *her9* mRNA is only weakly detected in homozygous mutants.** (A) *her9* heterozygotes were intercrossed and fixed at 24 hpf. *In situ* hybridization detected strong *her9* expression in wild types and only weak expression in *her9* mutants. (B) *In situ* hybridization detects broad *her9* expression in the head at 48 hpf in wild-type animals.

**SUPPLEMENTARY FIGURE 4: The cartilage craniofacial skeleton is smaller in *her9* mutants compared with wild types**. *her9* heterozygotes were intercrossed and fixed at 6 dpf and stained with Alcian Blue and Alizarin Red. Genotyped homozygous wild type (n=9) or homozygous *her9* mutants (n=4) animals were imaged in whole mount and the length of the symplectic cartilage and the ceratohyal were measured on both the left and the right side and summed. Cartilage measurements are presented as box and whisker plots, and the box extends from the 25th to 75th percentiles. The line in the middle of the box is plotted at the median, and the bars are minimum and maximum values. We used a t-test to compare total symplectic and ceratohyal cartilage (left plus right sides) between genotypes.

**SUPPLEMENTARY FIGURE 5: Pharmacological Notch inhibition does not phenocopy *her9* mutants, but early inhibition does phenocopy *jag1b* mutant phenotypes.** Wild-type embryos were treated with DBZ to inhibit gamma secretase cleavage of Notch during the indicated time windows. Early treatment during the pharyngula period produced craniofacial phenotypes similar to *jag1b* mutants. Treatment during the hatching period produced body curvature phenotypes. Treatment during the early larval period did not overtly affect development. None of these treatments produced mineralization phenotypes like those found in *her9* mutants. Scale bars are 50 μm

**SUPPLEMENTARY FIGURE 6: *jag1b*;*her9*;*her6* triple homozygous mutants display general developmental defects and delay.** Genotyped triple homozygous mutants from the experiment described in Fig. 9 were imaged in whole mount. Arrows indicate severe heart edema.
