# Supplementary figures and images for: Distinct and redundant roles for zebrafish her genes during mineralization and craniofacial patterning
Source: Front Endocrinol (Lausanne). 2022 Dec 12;13:1033843. doi: 10.3389/fendo.2022.1033843 (PMC9791542; doi:10.3389/fendo.2022.1033843)

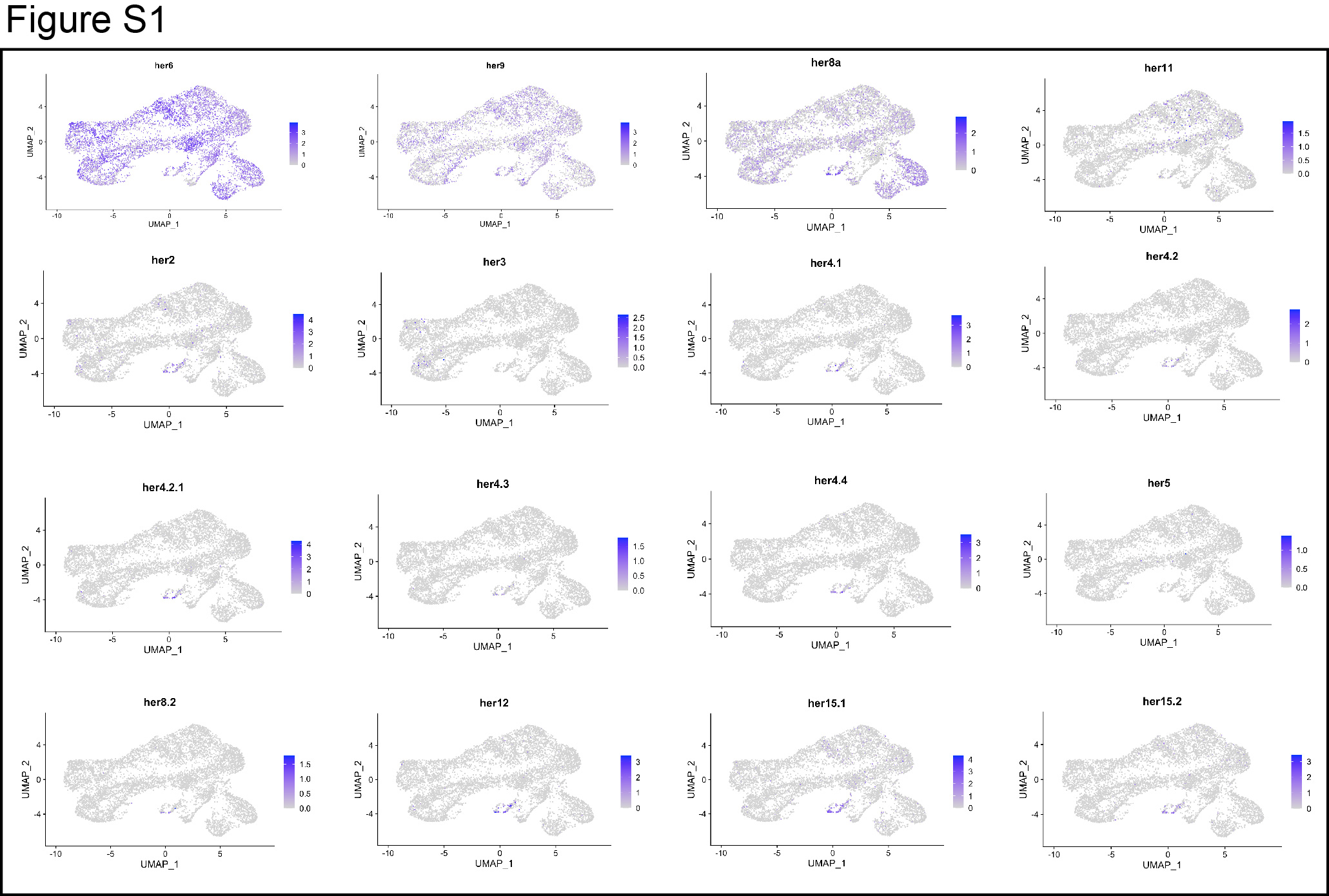

Supplement: Supplementary file 2 [file Image_1.jpg]

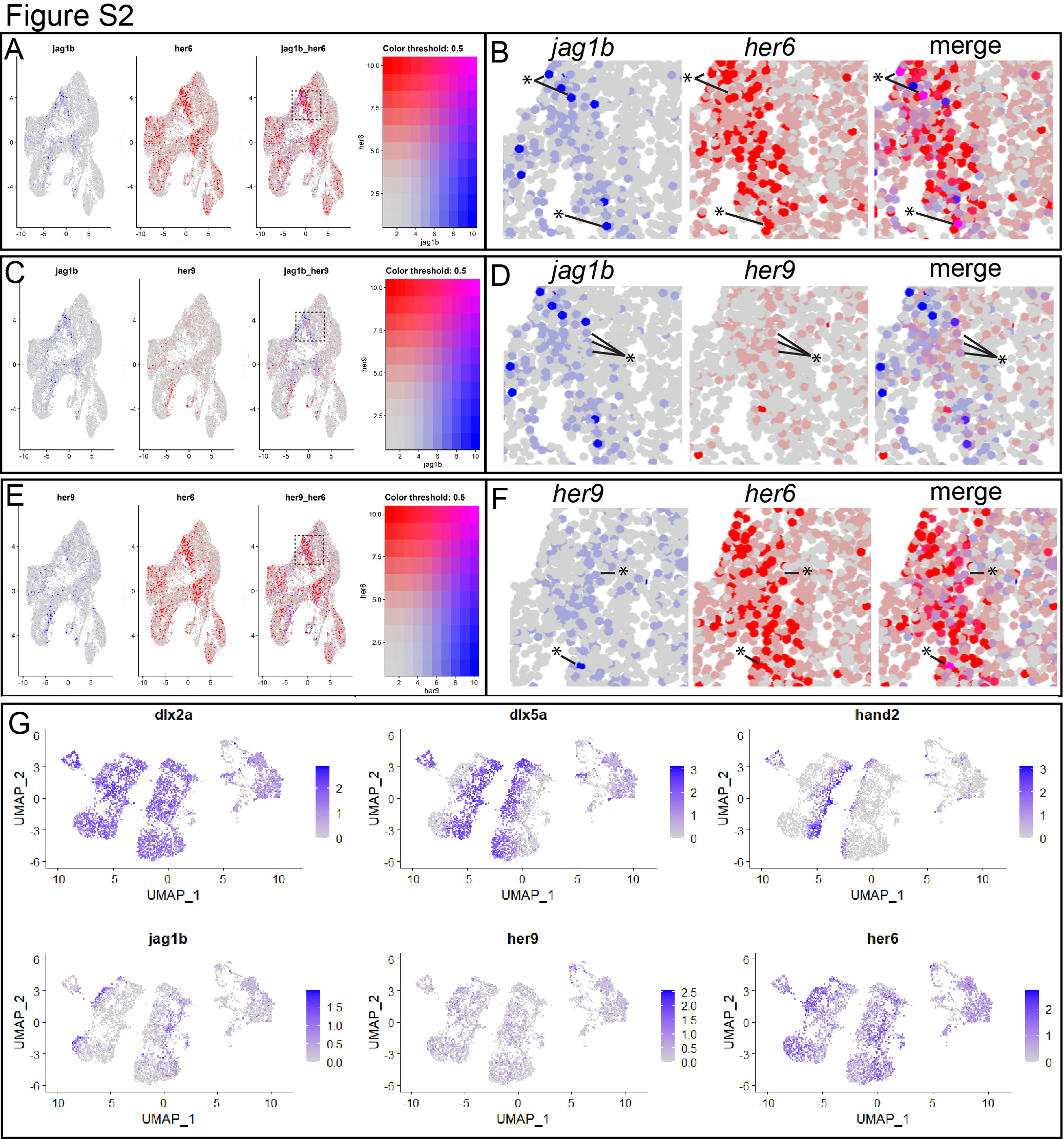

Supplement: Supplementary file 3 [file Image_2.jpeg]

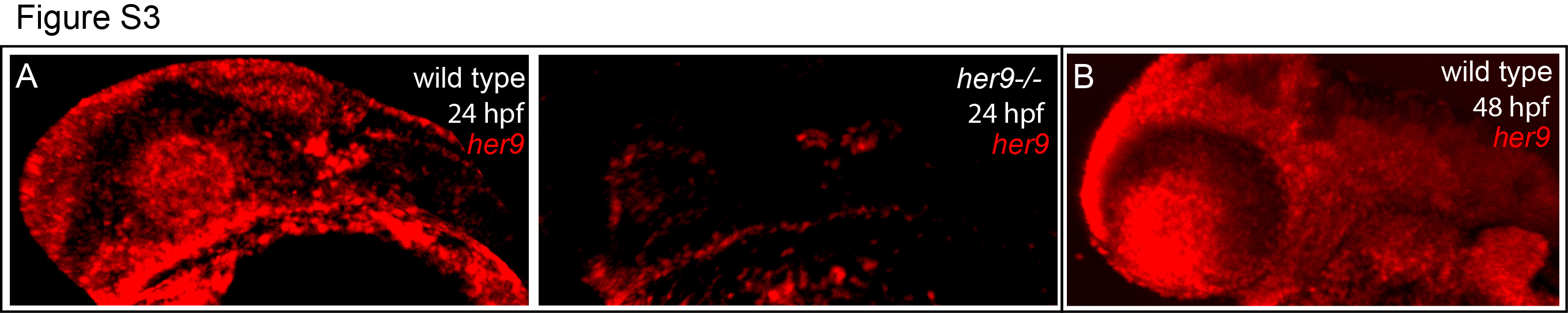

Supplement: Supplementary file 4 [file Image_3.jpeg]

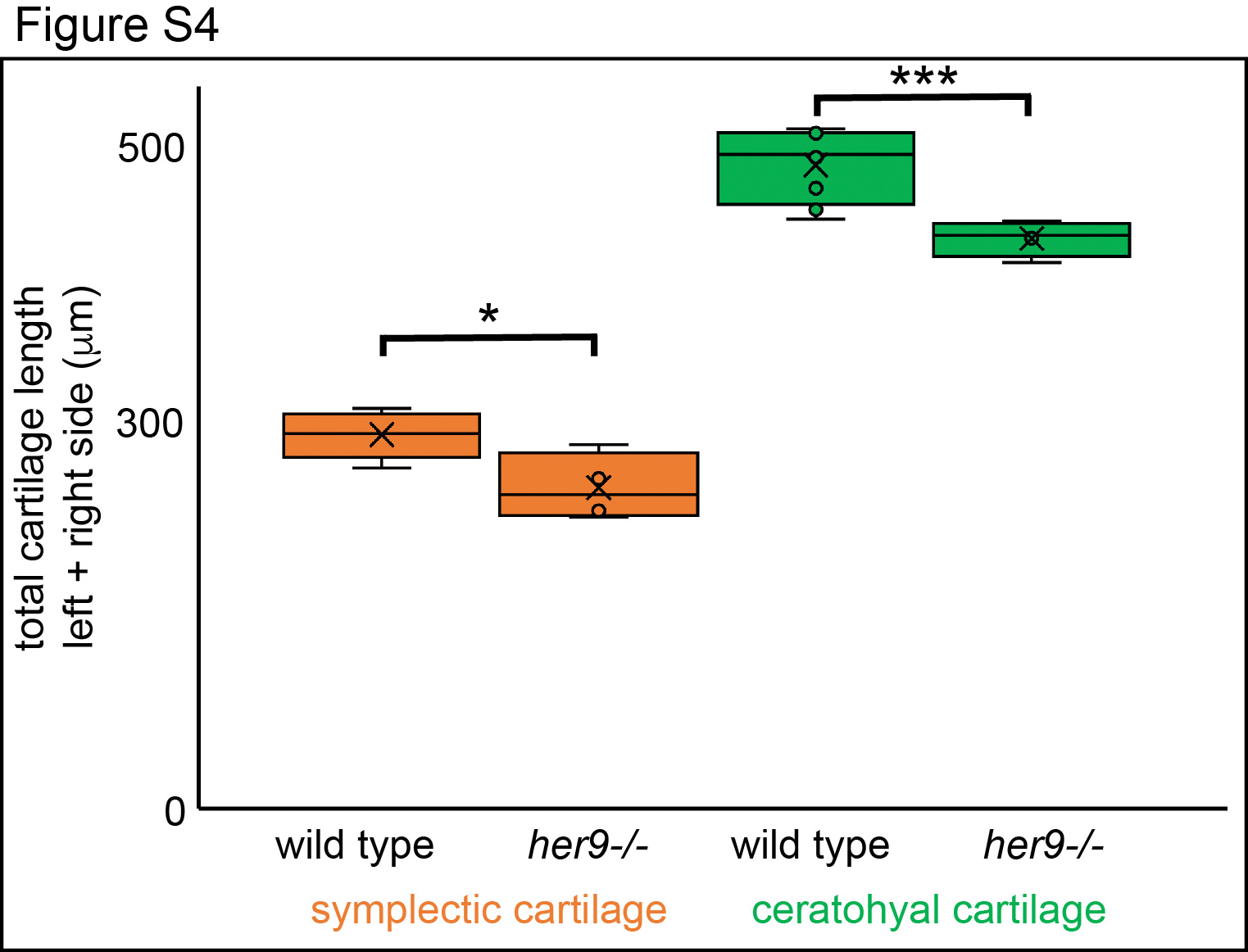

Supplement: Supplementary file 5 [file Image_4.jpeg]

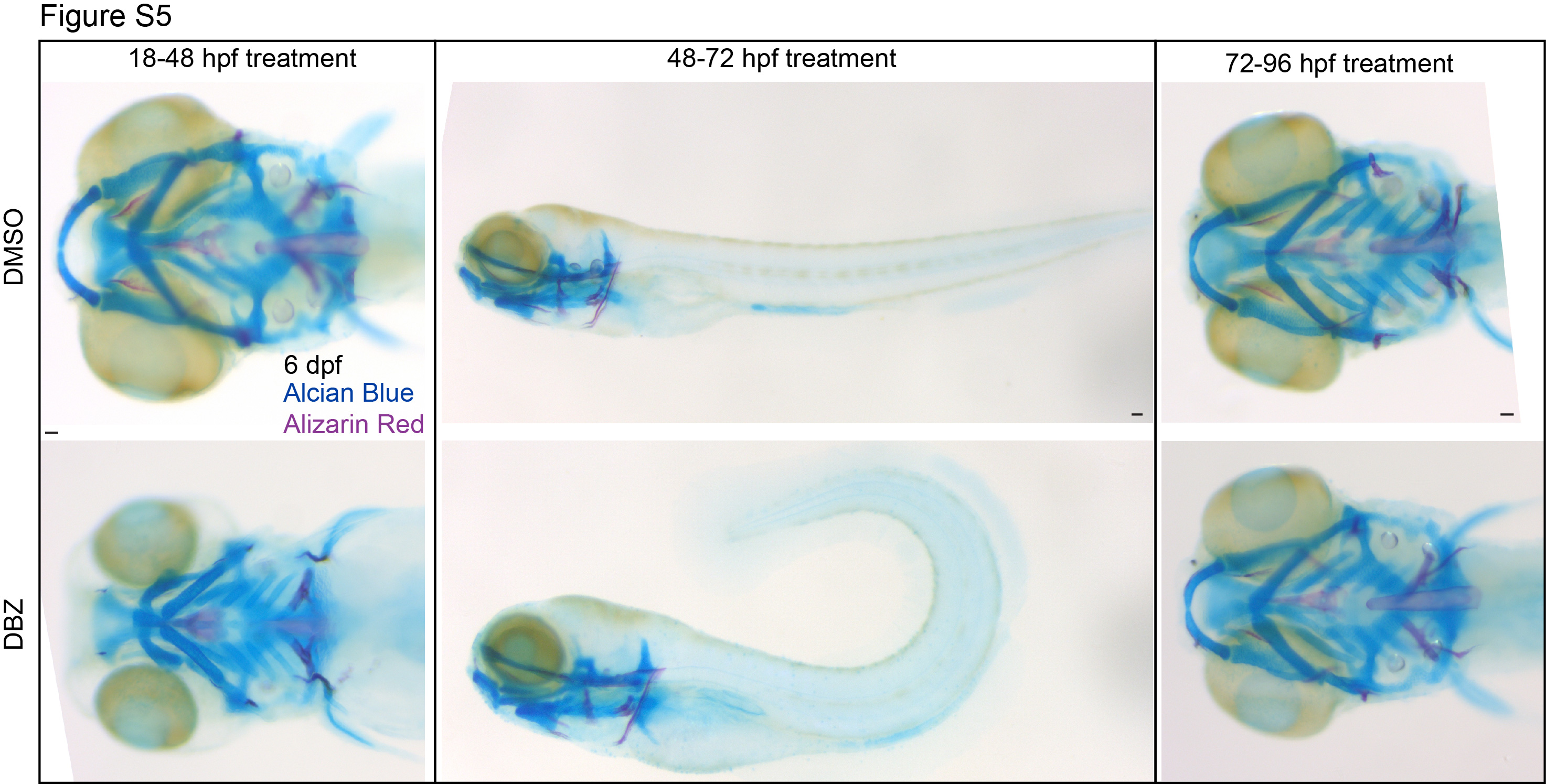

Supplement: Supplementary file 6 [file Image_5.jpeg]

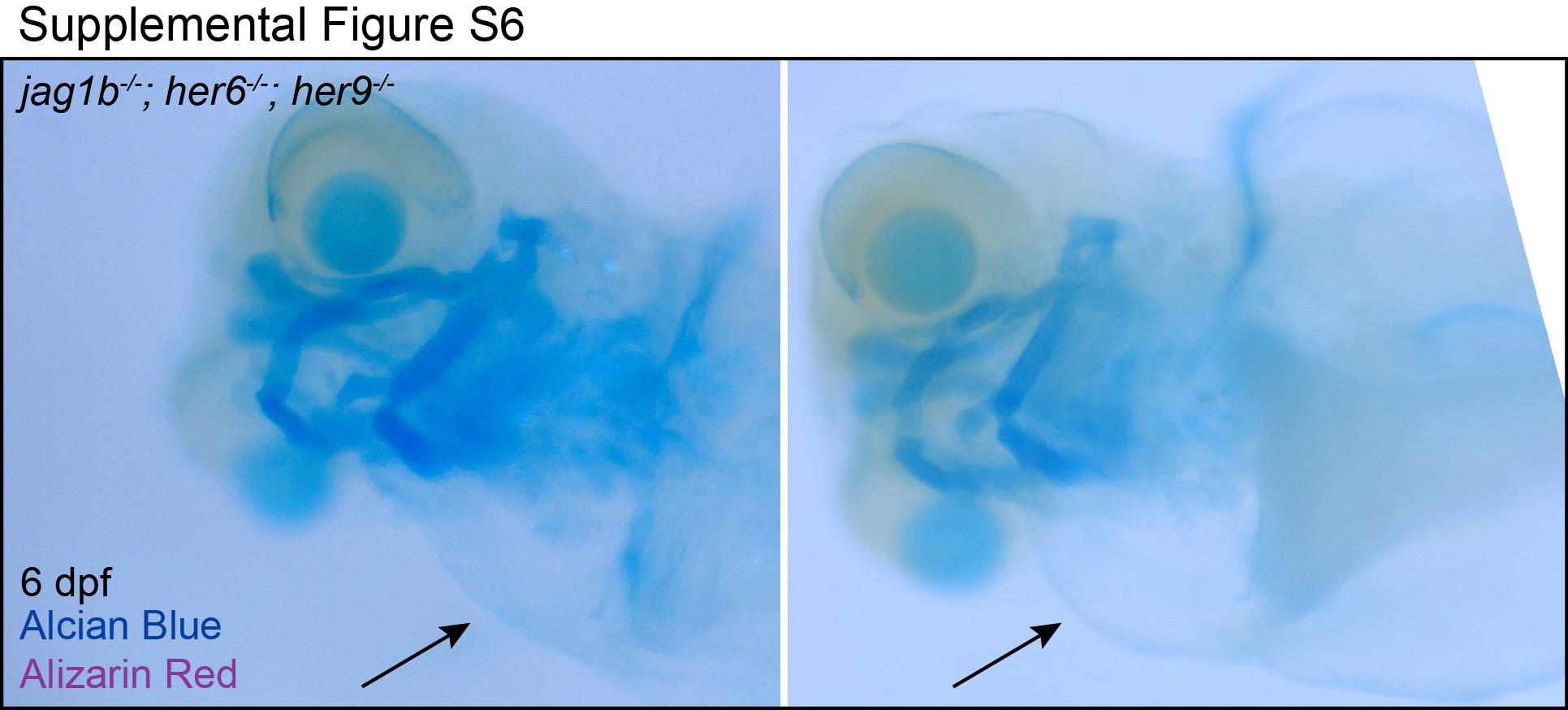

Supplement: Supplementary file 7 [file Image_6.jpeg]
